# Rapid Sampling of Molecular Motions with Prior Information Constraints

**DOI:** 10.1371/journal.pcbi.1000295

**Published:** 2009-02-27

**Authors:** Barak Raveh, Angela Enosh, Ora Schueler-Furman, Dan Halperin

**Affiliations:** 1 Department of Molecular Genetics and Biotechnology, Institute of Medical Research, Hadassah Medical School, The Hebrew University, Jerusalem, Israel; 2 School of Computer Science, Tel-Aviv University, Tel Aviv, Israel; Stanford University, United States of America

## Abstract

Proteins are active, flexible machines that perform a range of different functions. Innovative experimental approaches may now provide limited partial information about conformational changes along motion pathways of proteins. There is therefore a need for computational approaches that can efficiently incorporate prior information into motion prediction schemes. In this paper, we present *PathRover,* a general setup designed for the integration of prior information into the motion planning algorithm of rapidly exploring random trees (RRT). Each suggested motion pathway comprises a sequence of low-energy clash-free conformations that satisfy an arbitrary number of prior information constraints. These constraints can be derived from experimental data or from expert intuition about the motion. The incorporation of prior information is very straightforward and significantly narrows down the vast search in the typically high-dimensional conformational space, leading to dramatic reduction in running time. To allow the use of state-of-the-art energy functions and conformational sampling, we have integrated this framework into Rosetta, an accurate protocol for diverse types of structural modeling. The suggested framework can serve as an effective complementary tool for molecular dynamics, Normal Mode Analysis, and other prevalent techniques for predicting motion in proteins. We applied our framework to three different model systems. We show that a limited set of experimentally motivated constraints may effectively bias the simulations toward diverse predicates in an outright fashion, from distance constraints to enforcement of loop closure. In particular, our analysis sheds light on mechanisms of protein domain swapping and on the role of different residues in the motion.

## Author Summary

Incorporating external knowledge into computational frameworks is a challenge of prime importance in many fields of biological research. In this study, we show how computational power can be harnessed to make use of limited external information and to more effectively simulate the molecular motion of proteins. While experimentally solved protein structures restrict our knowledge to static molecular “snapshots”, a vast number of proteins are flexible entities that constantly change shape. Protein motion is therefore intrinsically related to protein function. State-of-the-art experimental approaches are still limited in the information that they provide about protein motion. Therefore, we suggest here a very general computational framework that can take into account diverse external constraints and include experimental information or expert intuition. We explore in detail several biological systems of prime interest, including domain swapping and substrate binding, and show how limited partial information enhances the accuracy of predictions. Suggested motion pathways form detailed lab-testable hypotheses and can be of great interest to both experimentalists and theoreticians.

## Introduction

Mechanistic understanding of protein motions intrigued structural biologists, bio-informaticians and physicists to explore molecular motions for the last five decades. In two seminal breakthroughs in 1960 [1],[2], the structures of Haemoglobin and Myoglobin were solved and consequently, for the first time, mechanistic structural insights into the motion of a protein were deduced from its snap-shot image. This finding paved the way to a by-now classical model for cooperativity in binding of allosteric proteins [3]. Nowadays, hundreds of proteins with known multiple conformations, together with their suggested molecular motion, are recorded in databases such as MolMovDB [4]. This number increases with the influx of solved structures from the Protein Data Bank [5]. An inherent flexibility is characteristic of fundamental protein functions such as catalysis, signal transduction and allosteric regulation. Elucidating motion of protein structures is essential for understanding their function, and in particular, for understanding control mechanisms that prevent or allow protein motions. Understanding the relation between protein sequence and protein motion can allow *de-novo* design of dynamic proteins, enhance our knowledge about transition states and provide putative conformations for targeting drugs. Accurate prediction of protein motion can also help address other computational challenges. For instance, Normal Mode Analysis (NMA) motion predictions [6] can be used for efficient introduction of localized flexibility into docking procedures [7],[8].

### Experimental Limitations and Progress

Experimental knowledge of macro-molecular motions has been discouragingly limited to this day by the fact that high-resolution structures solved by X-ray crystallography are merely the outmost stable conformations of proteins, in a sense a snap shot of a dynamic entity. While high resolution experimental data of molecular motion are still beyond reach, innovative breakthroughs in time-resolved optical spectroscopy, single molecule Förster resonance energy transfer (FRET), small-angle X-ray scattering (SAXS) [9], as well as advances in NMR spectroscopy such as residual dipolar coupling methods and paramagnetic relaxation enhancements [10]–[13] now provide increasingly detailed experimental data on molecular motion, e.g., distance and angle constraints or measurements of rotational motion [14].

### Computational Simulation of Motion

In spite, and perhaps due to the limited amount of experimental information, computational techniques like molecular dynamics (MD) simulations [15],[16] have been used extensively for the last three decades to simulate macro-molecular motion. Unfortunately, standard MD simulations are computationally intensive, and moreover, they often remain trapped in repetitive cycles of Brownian motion throughout the simulation, without being able to cross significant energy barriers. Therefore, they are often limited to pico-to-nano second timescales of motion [17], whereas events like enzyme catalysis [18], protein folding [19] and protein recognition [20] may require more time. As researchers often possess intuition and explicit partial knowledge about the nature of a motion or target conformations, biasing techniques were devised in steered MD simulations [21]. Such methods incorporate prior knowledge or expert intuition about the system and compromise the intended purity of MD simulations as a physical simulation. Nonetheless, they still rely to the most part on an approximation of physical forces, and guarantee that some plausible assumptions are satisfied. Subsequent motion trajectories were shown useful for designing experiments and deriving mechanistic insights into protein motion. Complementary coarse-grained methods such as Normal-Mode Analysis and Gö models [6],[22],[23] (reviewed in [10]) provide quick impressions about protein conformational changes when given a native conformation, but do not aim at the very fine details of the motion.

### Sampling-Based Approaches

In recent years, a novel approach for sampling motion pathways, rooted in algorithmic robotics motion planning, has been applied to large-scale molecular motion prediction. This approach suggests an efficient alternative to slow step-by-step simulations of Newton equations. Instead, a sequence of clash-free conformations is generated by sampling the topology of the conformational space. This sequence is a fine discretization of continuous motion. In their original context, motion planning techniques like probabilistic road-maps (PRM) [24], Rapidly-exploring Random Trees (RRT) [25],[26] and similar methods [27]–[29] (all reviewed in [26],[30]) have been used to plan the motion of objects with many degrees of freedom (*dof*s) among obstacles in a constrained environment [31]. (Usually, these objects are referred to as “robots”, but can be any moving object, such as digital avatars, manufactured parts, or molecules in the context of this study). For simplicity, we collectively refer to this family of techniques as motion planning sampling techniques.

In molecular biology, motion planning techniques were used to predict motion pathways for molecules while considering a large numbers of *dof*s [32]–[39], and contributed to our understanding of molecular kinetics in applications such as energy landscape exploration, protein and nucleic acids folding pathways and ligand binding [32], [34]–[36],[40]. Their performance has been compared to molecular dynamics [36] and integrated with Normal Mode Analysis [38]. In several cases, they were shown to capture known conformational intermediates and other experimental indicators [33], [37]–[39].

Motion planning techniques are optimized for finding complete motion pathways. They record the history and approximate the topology of the sampled search space in a tree or a graph data structure, the “road-map”. Molecular motions are extracted from paths or “roads” in the graph, where nodes stand for feasible (low-energy) conformations and edges connect close-by conformations. Therefore, paths in this graph are sequences of clash-free conformations. This also adds a whole new dimension of memory to the sampling process and the resulting search in conformation space is shown to be less prone to futile repetitive sampling [25].

Motion planning techniques are very fast – it takes between minutes and hours to generate a full motion pathway of relatively large time-scales with dozens of *dofs* and hundreds of amino-acids [38],[39], compared to weeks to months in MD simulations of motions with shorter time-scales. Hence, in contrast to MD simulations, sampling based methods are fast enough to generate a very large number of alternative pathways, whereas in an MD simulation it is often hard to decide if the pathway is representative or just the outcome of specific random start conditions. As the application of motion planning techniques to molecular motion is relatively new, further research is required in order to validate and calibrate its use. The external incorporation of experimental measurements into sampling-based simulations can increase the credibility of predictions, and turn them into a fair complement to *ab-initio* simulations.

In addition, as the dimensionality of the search space increases, it is advantageous to exploit prior information about the nature of the motion to direct the search. A common practice in sampling methods of single conformations like MC is to bias the energy function itself towards known constraints [41]. In the context of sampling-based motion planning, it is common to explicitly bias the sampling to include the target conformation (e.g. [42],[43]). Another common bias is towards narrow passages in the space of configurations [26],[44]. In order to avoid getting stuck due to over-bias, biased sampling is often restricted to a fraction of the tree growth iterations. Kalisiak and Panne [45] terminated RRT branches that lead to immediate collisions, by sensing the local environment on-the-fly in order to save running time. Zucker *et al.* [46] used various features of the workspace environment (the Cartesian representation of the world) to bias the sampling of motion planning algorithms, by introducing ad-hoc relations between robotic *dof*s and workspace features, and using a grid discretization of the workspace.

### Our Contribution

**Figure 1:**
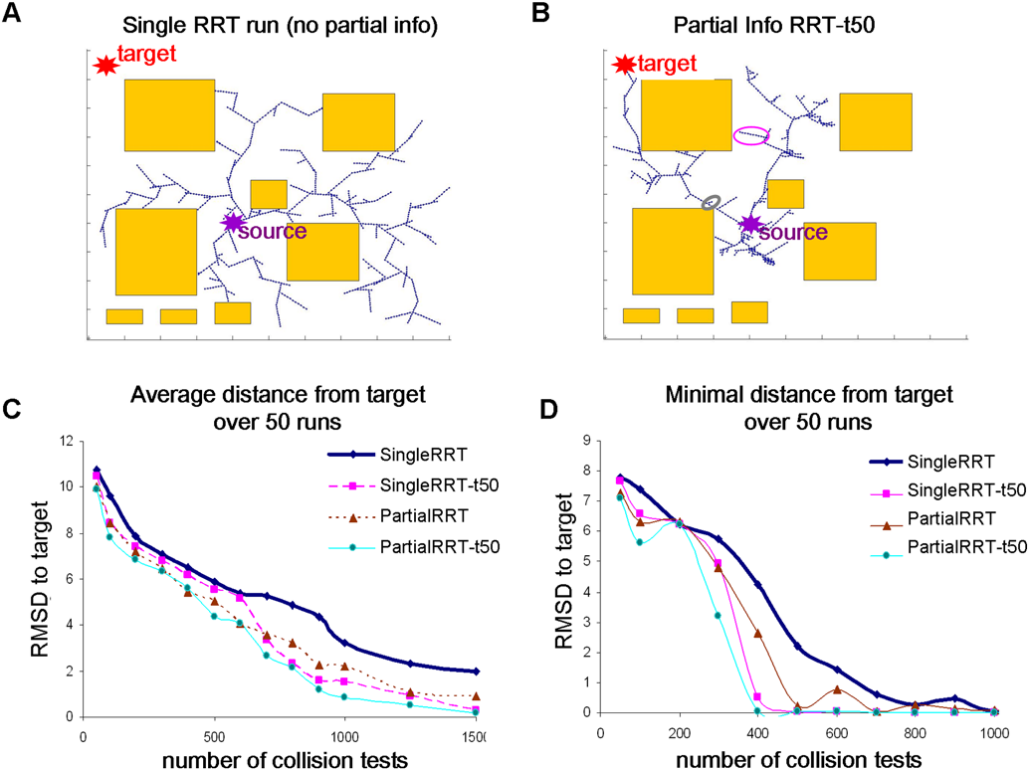
Comparison of pathway motion predictions in a 2D toy example. Here, we aim to find collision-free paths for a point robot in 2D-space starting from a source configuration. (A) The basic *Single-RRT* algorithm provides fast but rough coverage of unexplored regions, and the target is often missed (red star, top left). During the run, the tree grows in feasible space (white) among obstacles (orange rectangles). In biological examples, these obstacles are high-energy conformations. Each point stands for a two dimensional conformation, and the tree grows from a source conformation (violet star, middle of figure), towards random directions (see Methods). (B) In the *Partial-RRT* variant, we use partial information to truncate branches that do not grow towards the target (like the truncated branch in the grey ellipse, compare to the branch in the magenta ellipse). The search is more confined to relevant regions, at the expense of overall coverage of the search space. (C, D) Comparison between the basic *Single-RRT* algorithm and RRT with partial information (*Partial-RRT*), for the toy example in *a* and *b*. The partial information used here is the distance to the target. In *SingleRRT-t50* and *PartialRRT-t50* the target is also used as an explicit direction of growth once in 50 iterations, in case the tree reaches the proximity of the target but not its exact location. This test follows a common assumption that RRT running time is dominated by the number of collision tests. We compare the Euclidean distance of the RRT node that is closest to the target (y-axis) as a function of the number of collision tests (x-axis) throughout the run. Results are the average distance (in *c*) or the minimum distance (in *d*) over 50 independent runs. PartialRRT performed better than SingleRRT, especially for a lower number of collision checks. Better performance is achieved in less running time. As the number of collision checks grows. All methods converge. Note that this is only a toy example for illustrative purposes; in many biological examples, the target conformation might not be given explicitly, and the number of *dof*s is in general much higher.

Here, we present PathRover, a comprehensive and generalized framework for efficiently sampling and generating motion pathways that satisfy constraints of prior information with the RRT algorithm [25]. PathRover generates low-energy, clash-free motion pathways that are biased towards external constraints. This is in analogy to similar approaches for finding a single optimal structure (but not a motion pathway) under a set of experimental constraints [47],[48]. Our approach follows the notion that the combination of a number of partial constraints can significantly limit the number of feasible solutions. We rely on a generalized RRT formalism that allows efficient, flexible and straightforward integration of prior information into the basic RRT algorithm. Partial information is incorporated through a *branch-termination* scheme where the growth of undesired pathways from the RRT tree is terminated (see Figure 1 for a toy example that illustrates the effect of constraints on RRT motion sampling). To our knowledge, this is the first thorough generalized attempt to incorporate diverse types of prior biological information into the RRT algorithm in biological context.

We examine how limited geometric constraints can guide different types of motion towards a correct conformation. We deal with 8 to 198 backbone torsions, and model flexibility for all side-chain rotamers. We are motivated by the progress in experimental methods for extracting transient and non-transient distance constraints [10], e.g. using “experimental rulers” such as FRET and site-directed spin labeling experiments, or dynamic experimental measurements of the relative orientation of secondary structures [14] (Table 1).

**Table 1:** Examples for predicates of partial information in PathRover, motivated by experimental techniques and comparative methods.

Name of Predicate	Formal Definition of Predicate	Motivating Examples of Relevant Partial Information
***Pair Distance***	The distance between a pair of residues	Experimental distance constraints for transient and non-transient interactions (*Spin-Label NMR, Single-Molecule FRET, Cross-Linking*)
***RMSD Minimize***	RMSD between Cα atoms of two conformations or subdomains	Structure of an alternative native or homologue structure; the conformation of an active site region
***Line-Fit Distance/Angle***	The distance, or angle, between a set of Cα atoms, fitted by a least mean square line (LMSL)	Pairing of two beta strands ; relative orientation between the main axes of a helix and a sheet
***Cent-Mass Distance***	The distance between the center of mass of two subsets of Cα atoms	*Cryo-electron Microscopy* images that indicate the coarse distance between centers of mass of internal domains
***H-Bond Formation***	The formation of hydrogen bonds in unspecified locations	*Circular Dichroism (CD) spectroscopy* indications for helix-sheet formation, without indication of their specific location within the protein sequence

PathRover is integrated into the Rosetta molecular modeling framework [49], an accurate protocol for a range of different structural modeling tasks (e.g., [50]–[53]). Thus, PathRover is equipped with state-of-the-art energy functions, sampling and optimization protocols. All generated motion pathways are guaranteed to form a sequence of clash-free low-energy conformations, and to satisfy the input constraints.

### Model Systems

**Figure 2:**
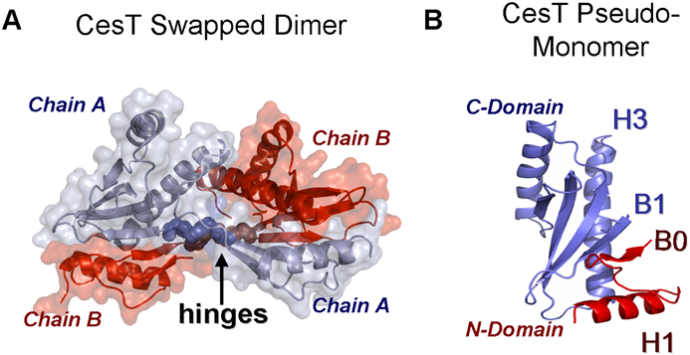
Domain swapping in *CesT* type III secretion chaperone. (A) Crystal structure of the swapped dimer, pdb-id *1k3e* [66]. The domains of each chain are packed against the complementary domains in the other chain. The presumed hinge region between the two domains of each chain is marked in space-fill representation. (B) The pseudo-monomer consists of the C-terminal domain from chain A (blue), packed against the N-terminal domain from chain B (red). Note the β-sheet in the interface between the two domains.

We mainly focus on domain swapping of two molecular model systems, the *CesT* and the *Cyanovirin-N* proteins. Domain swapping occurs in multi-domain proteins, when a domain from one chain packs against the complementary domain in an identical chain [54], forming a *pseudo-monomer* (Figure 2 and Figure S1). The *pseudo-monomer* resembles the native structure, and the interface between the swapped domains is native-like. Domain swapping can lead to undesired effects of aggregation, such as the formation of amyloidal fibrils [55]. Investigation of domain unpacking and repacking may improve our understanding of the general mechanism of oligomerization [56].

Domain swapping is an interesting target for motion simulations [57]–[60]. It requires the unpacking of domains in the original chain, and the subsequent repacking to another chain. The main structural changes between swapped conformations are usually restricted to a few hinge residues that connect the two domains [54]. This may allow for some simplifying assumptions about the degrees of freedom that are involved in the motion. Since the structure of each domain is identical in different conformations, we may assume they remain rigid during the motion. We examine the validity of this simplifying assumption and experiment with various choices of *dof*s. A clever choice of *dof*s may reduce the running time, but may introduce additional bias to the motion. We compare restricted runs where only a subset of torsion angles is allowed to rotate, to free runs where all degrees of freedom are mobile.

We note that the implications of the domain swapping examples are far reaching, since a large set of conformational motions is presumed to involve hinge motions with similar characteristics [61]. As an example, we consider the substrate binding motion of the Ribose Binding Protein.

PathRover supports full-atom simulations in which the output conformations contain the coordinates of all side-chains and hydrogen atoms. These conformations can be used to formulate precise, lab-testable hypotheses (e.g., suggest mutations that may interfere with the motion), which are of substantial interest to both experimentalists and theoreticians. In the following sections, we provide a detailed analysis of these models.

## Methods

### Conformational Space

The conformation space is described in terms of internal coordinates. Backbone torsion angles uniquely define the conformation of a protein, since the side-chain torsion angles are optimized on the fly for each given backbone conformation. Bond lengths and angles are fixed, assuming that changes in torsion angles can in general compensate milder changes observed in bond angles and length.

### Partial Information Predicates

We are interested in finding a collision-free, low-energy motion pathway that starts from a given initial conformation, and is consistent with partial information about the motion or the target conformation. We have formulated diverse types of predicates to constrain the sampling of motion pathways according to prior information. Here, we focus on partial information motivated by experiments, comparative analysis and expert intuition. For example, comparative analysis of biological databases can provide partial information from homologue structures, or from alternative conformations of the native protein, and distance constrains can be extracted from time-resolved spectroscopy. Table 1 includes a list of examples for predicates that are motivated by existing or yet to improve experimental methods for assessing transient conformations. Importantly, different types of partial information can be combined into a joint predicate. We note that distance constraints and additional constraints have been previously used in Rosetta to direct Monte-Carlo with Minimization sampling, although in a different algorithmic and biological context (see Discussion).

As the combinatorial search space grows exponentially with the number of *dof*s, it is also beneficial to restrict the choice of flexible torsion angles. An automated, accurate choice of mobile *dof*s is a challenging aspect of motion prediction, and in this step, prior information can be most useful (see [38] for an attempt in this direction). In this work, we have combined information from several sources for restricting the number of *dof*s, such as: (1) careful inspection of structures, (2) relevant literature, (3) computational tools for detecting hinge regions like Normal Mode Analysis (NMA) [22], and (4) comparison of structural changes in alternative (native or homologue) conformations. When both a source conformation and a target conformation are available, we used the FlexProt flexible alignment tool [62] to extract fixed regions of the protein, and defined the *dof*s by the regions in-between. These were used to manually restrict the allowed *dof*s in the examined model systems (Table 2). The effect of the choice of mobile degrees of freedom is examined in detail in the Results section.

**Table 2:** Backbone degrees of freedom (*dof*s) that were free to move in simulations. For each model system we include the main evidence that was used for choosing a specific set of degrees of freedom.

Name of Simulation	Mobile Residues	Evidence Used for Selection of Residue Degrees of Freedom
*CesT* [M]^1^^,^ 2	**34–37** *(8 dofs)*	• N-terminal domain (residues 1–33) and C-terminal domain (residues 38–134) can be independently aligned to homologue counterparts (e.g., SigE) by a rigid transformation. The pseudo-monomer is obtained by the packing of domains A and B.
		• Two slowest modes of GNM^3^ analysis predict hinges at residues 34 and 37.
		• Manual inspection shows that residues 34–37 side-chains are unpacked, and are flanked by two well-packed domains with regular secondary structures.
*Ribose Binding Protein* (RBP)^4^	**101–104**; **234–236**; **261–262** (*18 dof*s*)*	• Extract loop residues that connect the two structured domains based on manual inspection (Figure 4a).
		• Each structured domain is structurally conserved between conformations 1urp and 2dri.
		• Slowest mode for GNM^3^ of pdb-id 2dri predicts hinges at residues 103–104, 235–236, 262–265, in the vicinity of the selected degrees of freedom.
*Cyanovirin-N* 5: Central-Hinge	**48–55** *(16 dofs)*	• N-terminal domain (residues 1–50) and C-terminal domain (residues 51–101) are repeat domains at the sequence and the structure level (<1 Å RMSD deviation). The structure of each domain is highly conserved between alternative conformations, but not that of the connecting residues 48–55.
		• Large differences in φ/ψ values between alternative structures 2ezm, 1l5b and 1l5e around this approximate region.
		• The literature about Cyanovirin-N structure marks this region as the hinge region [72],[73].
		• Mutations of P50 and S51 significantly affect the equilibrium between the monomeric and dimeric forms [73].
		• Slowest mode of GNM^3^ analysis for pdb-id *1l5e* predicts hinges at residues 50–52.
*Cyanovirin-N* 5 *:* Secondary-Hinges [M]^2^	**48–55**; **36–40**; **87–91** *(32 dofs)*	• Secondary hinge residues 36–40 and 87–91 connect separate secondary structures within the N-terminal and C-terminal domain, respectively.
		• GNM^3^ analysis: slowest/second-slowest modes for pdb-id *2ezm*, and second slowest mode for pdb-id *1l5e*, both predict hinges around residue 34–36 and 86–87, in the vicinity of the selected degrees of freedom.
*Cyanovirin-N* 5 *:* Partially-Restricted [M]^2^	**48–55**; **2–47**; **56–100** *(198 dofs)*	Here we allow for “breathing motion” of ±30° in torsions 2–45, 56–100, in addition to full motion in the central hinge, like in the *central-hinge* simulation above (residues 48–55).
*Cyanovirin-N* 5 *:*Free [M]^2^	**2–100** *(198 DOFS)*	All torsion degrees of freedom (except extreme tail residues) are free to move by ±180°.

1 pdb-id used: *1k3e* [66] ; protein total length is 146 residues.

2 Simulations with local energy minimization are denoted by [M], see Methods.

3 Gaussian Network Models analysis on iGNM server http://ignm.ccbb.pitt.edu/ [22], using default cutoff parameter of 10 Å for building the harmonic potential.

4 pdb-ids used: *1urp* [83], *2dri* [84] ; protein total length is 271 residues.

5 pdb-ids used: *2ezm* [74], *1l5e* [73]; protein total length is 101 residues.

### RRT Motion Planning with Partial Information

The Rapidly-exploring Random Tree (RRT) algorithm is a general framework for rapid exploration of a conformation space (referred to as “configuration space” in robotics) in a highly constrained environment. It was first presented in algorithmic robotics, where it was used to plan the motion of moving objects among obstacles [25]. RRT produces a tree of conformations and records the topology of the search space. Nodes stand for feasible (low-energy) conformations, edges connect close-by conformations, and paths are sequences of feasible conformations. It was shown that the RRT tends to grow towards unexplored regions at progressively increasing resolution [25].

#### Forbidden space and feasible space

We define the conformation space by the internal *dofs* of the protein, namely the torsion angles that are allowed to change throughout the motion pathway (see below). The conformation space is divided into forbidden and feasible regions (referred here as *C-forbid* and *C-feasible,* respectively; for illustration see Text S1). The forbidden regions correspond to all the conformations that involve high energy values, namely energy score above a threshold parameter, whereas the feasible regions comprise the low-energy conformations.

#### RRT algorithm with branch termination

In algorithmic robotics literature, RRT is often biased by manipulation of node sampling, e.g. sampling in certain regions of interest. Here we take a different approach and rely instead on terminating branches that do not improve a certain predicate (note that branch termination has been previously used in a rather different context only, namely for avoiding imminent collisions under kinematic-dynamic constraints by sensing the immediate environment [45], and to deal with moving obstacles [42]). The input to the algorithm is an initial conformation and a set of partial information predicates (detailed pseudo-code and a full list of parameters are provided in Text S1). The tree is grown iteratively in small incremental moves to guarantee the smoothness of the motion. At each iteration, a new conformation, q_rand_, is randomly sampled from the feasible space *C-feasible*. The nearest neighbor in the tree is then expanded towards q*_rand_*_,_ by linear interpolation of the degrees of freedom from q_near_ to q_rand_. Each path in the RRT tree can be considered a fine discretization of a continuous motion pathway in the feasible conformation space. The simulation terminates when: (i) the number of nodes in the tree is larger than *N*, a parameter for maximal tree size, or (ii) the tree could not be expanded for *k* consecutive iterations. The partial information predicates are used to choose a motion path that leads from the initial conformation to the conformation with the best predicate score.

The partial information bias is introduced by a filtering step. The filtering step is applied only in every other iteration, to allow an escape from local minima traps. In the filtering step, the branch that grows towards q_rand_ is terminated if it does not improve the partial information predicate after *m* consecutive interpolation steps (typically *m* = 2, again to allow an escape from local minima traps). The branch is terminated even if it leads to energetically feasible conformations (Figure 1b). The aim of this filtering step is to avoid expensive energy calculations in undesired directions. We note that existing branches are not pruned, only the growth of the current branch is terminated.

#### The effect of avoiding local minima with respect to partial information

Figure 1 shows a toy example where branch termination is applied in all iterations. As desired, this narrows down the search to relevant regions of the conformational space (Figure 1b, grey circle). If we compare to Figure 1a, where branch termination is not employed, we see that the overall coverage of unexplored regions is compromised, but the target is reached faster (Figure 1c and 1d). By applying the global filtering step in every other iteration, we gain a bias towards partial information predicates, but still benefit from the rapid sampling of the unbiased RRT algorithm.

#### Side-chain optimization and local minimization

In full atom-mode (see below), side-chains of generated conformations along the pathways are locally refined by the Rosetta Rotamer-Trial procedure to alleviate local steric clashes and optimize the interaction of side-chains with neighboring residues [63]. In addition it is advisable for full-atom runs to include short gradient-descent minimization with respect to *all* torsion degrees of freedom: very slight rotations of torsion angles (∼0.1°–0.2°) can alleviate local steric clashes and reduce the energy score substantially. To restrict the change in φ/ψ values, we added a heavy Gaussian penalty for deviating from the initial backbone torsions (Rosetta energy constraint *CST_PHI_PSI* with weight = 250.0). Note however that minimization is time consuming (3–8 times slowdown). Local minimization is optional and can be invoked by turning on a run-time flag.

### Rosetta Infrastructure

#### Energy function: Full-atom vs. centroid mode

We experiment with both the Rosetta full-atom energy function (Rosetta score12 [50]), which was shown useful for discerning native structures at atomic detail, and the coarser Rosetta centroid-mode energy function, a united-atom representation where side-chains are represented as centroid spheres (Rosetta score4 [49]). The latter allows rapid calculations at the expense of atomic detail, and has been used in a wide range of applications in Rosetta to speed up the conformational search by optimizing coarse features prior to atomic level optimization.

We assume here that the Rosetta energy function is relevant for our current task (see Discussion). The Rosetta energy function was optimized for native structures, but it includes physical van der Waals terms and solvation models, as well as a statistical hydrogen bonding term that was shown to correlate with quantum mechanical calculations [64]. However, we do note that PathRover is in principle not restricted to any specific energy function, and can be used in conjunction with other energy potentials as well.

#### Rosetta optimizations

In addition to the full-atom and centroid-mode energy functions, the presented framework takes advantage of the elaborate infrastructure of Rosetta, including manipulation of molecular *dofs*, rapid side-chain optimization for fixed backbones (using the “rotamer trial” procedure described in [63]), energy minimization and caching of energy calculations. The framework can also use the gamut of other Rosetta features, such as closed loop sampling and sophisticated manipulation of backbone torsions, e.g. backbone fragment libraries or backrub motions [65]. These features are out of the scope of the current study, and will be explored in future work.

### Analyzing Hinge Residues in Simulations

In order to characterize the predicted motion in our simulations, we have examined what portions of proteins remained rigid during the motion and what residues served as hinge residues. We note that inspection of φ/ψ values is not necessarily suitable for this purpose, since small backbone perturbations can result in large scale motions and *vice versa*. In Figure S2, we describe our protocol for detecting hinge residues in simulated motion. In brief, we rely on structural comparison of different pairs of conformation in the simulated motion. Rigid portions of the protein are detected by the FlexProt [62] flexible structural alignment algorithm (Figure S2), and the hinge residues are defined as the regions that connect the rigid parts. We score each residue for how often it serves as a hinge in different alignments throughout the simulation (Figure S2). The structural alignment is performed at different resolutions of RMSD, using a resolution parameter ρ. Low resolution hinges are involved in strong hinge motions, and high-resolution hinges are involved in milder ones.

#### Running times

All runs in this study were conducted on an AMD Opteron 275 2.2 Ghz/1 MB processor. Unless otherwise specified, in the Results section, centroid mode runs take the order of a few seconds to minutes each and Full-atom runs take 2–8 hours without energy minimization, and roughly 10–60 hours when the energy minimization flag was employed (see above), for growing trees of 30,000–100,000 conformations each. The number of *dof*s in different runs was between 8 and 198 backbone torsions (and all side-chains, see Table 2).

## Results

In the first part of this section, we examine the usage of various geometric constraints and a combination of constraints to bias the motion during simulations. We also show how the energy function prevents over-bias by the input constraints. In the second part, we deal with another form of partial information – the choice of degrees of freedom that are allowed to move during the simulations. We examine the robustness of simulations to different choices of degrees of freedom, and analyze in full-atom detail the domain-swapping motion of inspected model systems.

### Partial-Information Predicates to Bias the Motion

#### CesT domain swapping

*CesT* is a type III secretion chaperone in *Enteropathogenic E. coli* that binds numerous effector proteins. In *CesT* the neighboring chains within the crystal lattice are domain swapped [66] (Figure 2a). The N-terminal domain (residues 1–33) and C-terminal domain (residues 38–134) from neighboring chains pack to form a monomer-like globular unit, the “pseudo-monomer” (Figure 2b). The pseudo-monomer can be well aligned to monomers in the other homologues (Figure S1), suggesting that there is a monomeric form of *CesT* that resembles the pseudo-monomer. Packing of non-swapped monomers against each other is mostly identical to their packing in the pseudo-monomers, as the sequence of domains is identical [56]. It is however not known whether the swapped conformation of *CesT* is a crystallographic artifact or whether it is the physiologically active peptide-binding form [66], and it is interesting to examine the possibility for domain-swapping motion of this protein.

#### Using the pseudo-monomer as a partial information predicate

We first examine whether we can model a hinge motion in which a chain of *CesT* moves to the pseudo-monomer conformation where its two domains are interacting. We start from the swapped conformation (where the domains are farther apart), using chain A in pdb-id 1k3e [66]. We allowed backbone mobility in residues 34–37, a loop region that separates the two domains and is also predicted to be a hinge region by normal mode analysis (Figure 2a, spheres; see also Table 2 for additional evidence that these residues form a hinge). The algorithm was biased to minimize the RMSD between the initial conformation and the pseudo-monomer conformation, using the residues in the interface between the N-terminal domain and the C-terminal domain (Table 3). We applied RRT with branch termination, as described in Methods, and simulated the motion in both centroid mode and full-atom mode (where all side chain atoms are included, see Methods). Each centroid mode run was repeated 50 times, taking a few seconds only to complete. Full-atom mode simulations were performed with the energy minimization flag turned on, to relieve local steric clashes. Each such simulation took a few hours on a single processor, and was repeated 15 times.

**Table 3:** Predicates used for guiding the domain swapping motion of CesT.

Name of Predicate	Description of Predicate	CesT Residues	SigE Residues
**Pseudo-monomer**	Minimize RMSD between CesT and the pseudo-monomer of the CesT crystal structure	S5:C29 Y38:N62	*not relevant*
**Atom distance A**	Compare the distance between a pair of atoms in CesT and in SigE. The distance in SigE is used as a reference for CesT during the simulation. A, B and C are three different choices of atom pairs.	F12:E110	L8:L95
**Atom distance B**	see Atom distance A	L8:A104	L4:S89
**Atom distance C**	see Atom distance A	D34:Y37	D27:I29
**Atom distance A+B**	Weighted combination of above Atom-Distance predicates: 1*A+1*B	F12:E110 L8:A104	L8:L95 L4:S89
**Atom distance A+B+C**	Weighted combination of above Atom-Distance predicates: 1*A+1*B+1*C	F12:E110 L8:A104 D34:Y37	L8:L95 L4:S89 D27:I29
**Helix line-fit**	Fit a least-mean square line (LMSL) to both helix H1 and H3. The predicate is a weighted sum of the three terms: 1*Line_angle+1*Line_dist+1*CMass_dist *Line_angle* = the angle between fitted lines *Line_dist* = the distance between fitted lines *CMass_dist* = the center of mass distance from helix H1 to H3	**Helix H1:** L8..K15 **Helix H3:** P106..L125	**Helix H1** L4..A11 **Helix H3:** E91..E110
**Helix RMSD**	Minimize RMSD between helices H1 and H3	Identical to **Helix Line-Fit**	
**Strand RMSD**	Minimize RMSD between β-strands B0 and B1	**Sheet B0/1:** A32..D34 I36..L41	**Sheet B0/1:** 23..25 29..34
**Helix+strand RMSD**	Minimize RMSD between both helices H1/H3, and sheets B0/B1 (with equal weights)	Identical to **Helix RMSD+Sheet RMSD**	

We analyzed the runs that best minimized the predicate. Both in centroid mode and full-atom mode, a collision-free path towards the pseudo-monomer conformation was found. The initial conformation deviates by 16 Å RMSD from the pseudo-monomer, and the final conformation deviates by 0.8 Å RMSD in centroid mode, and 1.34 Å in full-atom (0.76 Å for a partial alignment without residues N24-I33). These runs provide a proof of concept that the biased RRT algorithm successfully employs bias for guiding the motion. The suggested motion is shown in Video S1 (full-atom mode) and Video S2 (centroid side-chains mode).

#### Biasing the motion with SigE, a homologue of CesT

*SigE* (pdb-id *1k3s* [66]) is one of a few distant homologues of *CesT*. The N-terminal and C-terminal domains of *SigE* are similar to those of *CesT* (RMSD of 1.7 Å and 2.5 Å respectively), and the pseudo-monomer of *CesT* can be aligned to the *SigE* monomer (Figure S1). However, the two structures share a very low sequence identity (18%), and the *SigE* monomer deviates by 3.96 Å from the pseudo-monomer (using the sequence alignment from [66]).

**Figure 3:**
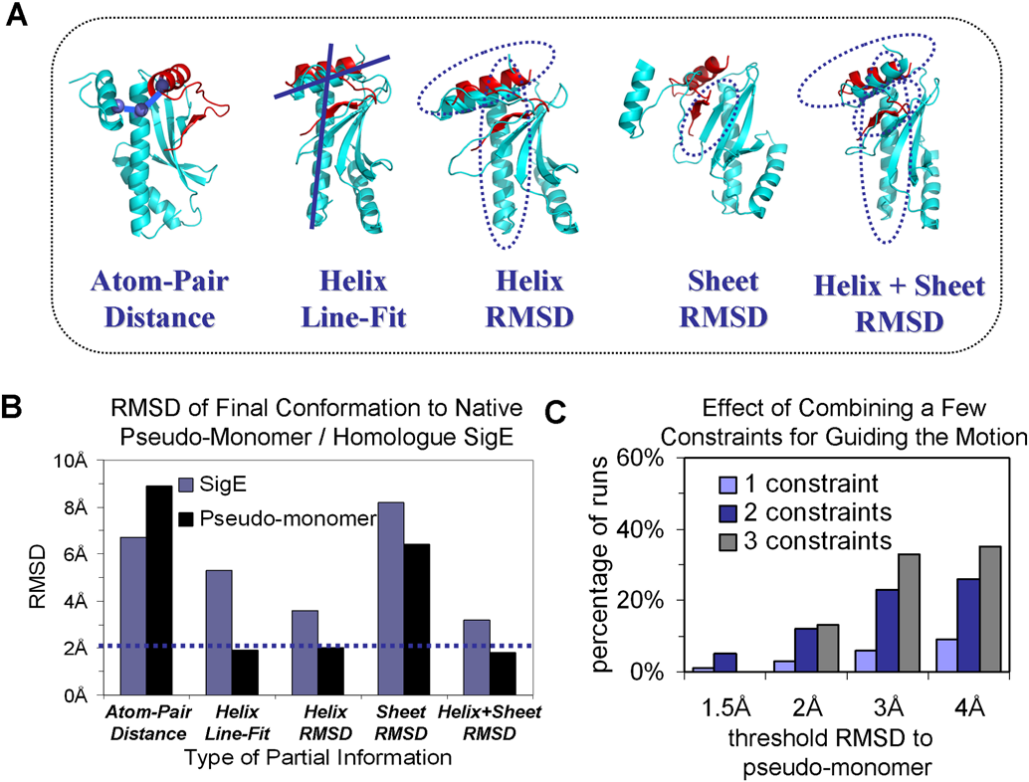
Use of partial information in simulations of *CesT* domain swapping (in centroid mode representation). (A) The final conformation along the motion pathway of *CesT* (cyan) is shown for five different examples of predicates (see Table 3). We show the best scoring run with respect to the specified predicate (out of 50 independent runs). The orientation of the N-terminal domain of the native pseudo-monomer is shown in red for comparison. (B) The RMS distance of the final structure, for simulations with different predicates, is plotted relative to *SigE* (the homologue that was used to guide the motion, blue) and the native pseudo-monomer of *CesT* (black). Even though the homologue was the reference for biasing the motion, the simulations reached the correct conformation with a better level of accuracy for several predicates. (C) Biasing the motion by combining several distance constraints (see Table 3 for details about the constraints): the results are shown as the fraction among 50 independent simulations that reached given RMSD thresholds (to the native pseudo-monomer).

In order to investigate whether this distant homologue can indeed guide the motion towards the correct conformation, we devised a set of geometric predicates that use SigE as a reference for guiding the motion of CesT, such as the distance between specific atoms or the orientation between specific secondary structures (see Table 3 and Figure 3). For each predicate, we conducted 50 independent runs and analyzed the run that best minimized the predicate. We worked in centroid (united-atom) mode, as this example mainly serves to illustrate the effect of various predicates on the simulations. Running time was a few seconds for each simulation.

In Figure 3a and 3b we examine, for 5 of the predicates in Table 3, the RMSD distance of the final conformation from (1) SigE – the reference homologue protein, and (2) the native CesT pseudo-monomer (Figure 2b). Remarkably, in four cases, the final structure was more similar to the pseudo-monomer than to SigE, even though the reference for guiding the motion was SigE (the SigE monomer deviates by 3.96 Å from the pseudo-monomer). This suggests that the energy function prevented over-bias of CesT motion towards the structural features of SigE. This fact is particularly surprising since the simulations were conducted in centroid mode, without the atomic details of the side-chains, and demonstrates the effectiveness of a simplified and rapid energy function in this case. At such level of predictive precision (<2 Å), many side-chains can be already modeled quite accurately. Although our aim is motion prediction and not homology modeling, it is promising that the near-native conformation is recovered using very simple predicates.

#### Relative orientation of secondary structures

In *Sig-E* and in the *CesT* pseudo-monomer (but not in the initial conformation), α-helices H1 and H3 each lie in a different domain of the protein (Figure 2b). In addition, β-strand B0 of the N-terminal domain is paired to β-strand B1 of the C-terminal domain (Figure 2b). In Table 3, we list the set of predicates that we formulated over the relative orientation of these secondary structures in the two domains. An interesting predicate is the Helix Line-Fit predicate, which combines three measures. We used least mean square line fitting (LMSLs, Table 1) to approximate the main axes of α-helices H1 and H3. We then measured the distance and angle between the fitted lines, as well as the distances between the centers of mass of each helix. This is a useful measure when relying on a homologue protein, since it is much less sensitive to alignment shifts that are characteristic for α-helices. In Figure 3b we observe that the final conformation was very close to the native pseudo-monomer (1.87 Å) but not to SigE, which is the reference for the biasing predicate. Looking closely at this example (Table S1), we saw that the line angle and line distance predicates were perfectly matched to their values in *SigE*, whereas the center of mass distance between the helices did not reach its value in *SigE (15 Å)* but rather reached its value in the CesT pseudo-monomer. Hence, the simulation was not over-biased by partial information constraints, and took into account the specific features of the simulated molecule.

#### β-sheet formation

The combination of helix and sheet predicates (Table 3 and Figure 3) was sufficient to direct the motion to the native conformation. How does β-sheet formation affect α-helix orientation and *vice versa*? We observed that when the motion was guided by partial information on the orientation of the α-helices alone, the β-strands B0 and B1 still came close together and the final conformation exhibited similar structure to the native pseudo-monomer. In contrast, the helices did not move to the correct orientation when the only partial information provided was β-sheet formation. We note however, that this may also be an artifact of the smaller number of atoms involved in the strand-pairing predicate.

#### Combinations of atomic distance constraints

Not surprisingly, a constraint on the distance between a single pair of atoms is not deterministic enough for guiding the motion (Figure 3a and 3b–left bar). The structural alignment between the final conformation of *CesT* and the pseudo-monomer is rather poor. It is clear that the distance between a single pair of atoms should be combined with other partial information or atomic detail constraints, in order to derive a more reliable target conformation and motion pathway. Therefore, we have examined what combination of distance constraints suffices for biasing the motion. Combinations of two or three distance constraints (Table 3) were used to guide the motion. In Figure 3c, we plot the percentage of 50 independent simulations that reached the native pseudo-monomer conformation up to various degrees of similarity (in RMSD). We observe that 2 or 3 constraints are still not enough to guide the motion in all simulations, but they lead to a much higher percentage of runs that reach the native conformation. This suggests that the combination of just a few distance constraints is an effective way of constraining motion-planning simulations.

#### Ribose binding protein (RBP): Ligand-binding-induced hinge movement: Incorporating loop closure constraints with simple predicates

The problem of fixing remote structural segments that are connected by a flexible loop is known in the literature as the protein loop closure problem [67]. It might require complex loop closure calculations or interpolation of internal coordinates motion from Normal-Mode Analysis [38],[68]. Previous attempts have been made for *ad-hoc* solutions to this problem during RRT simulations [68],[69], as well as in the broader context of structural modeling [67],[70],[71].

**Figure 4:**
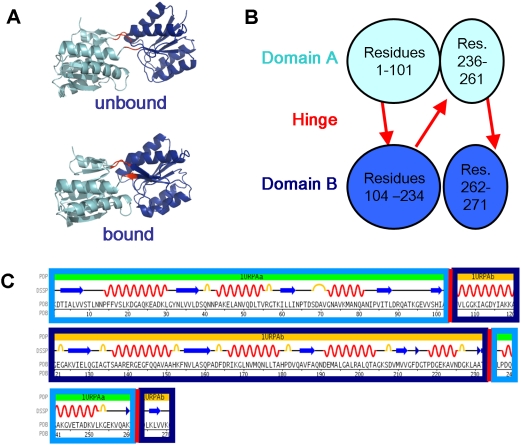
*Ribose Binding Protein (RBP)* architecture. Domain A (cyan) is connected to Domain B (blue) by a hinge (red). (A) The *RBP* structure in its open and closed form, pdb-ids *1urp* [83] and *2dri* [84] respectively. (B) The architecture of *RBP*–each domain consists of discontinuous segments of residues. The two domains are connected by three hinges that must move in a coordinated way to maintain domain integrity. Domain boundaries are rough estimates. (C) The sequence of *RBP* showing the discontinuous domains and the secondary structures. This illustration was taken from 1urp Protein Data Bank entry at http://www.rcsb.org/pdb/ [5]; and domain assignments are from [85].

The *Ribose Binding Protein (RBP)* belongs to a family of ligand-binding proteins that comprise two domains, connected by a hinge. Upon binding of the ligand in a cleft between the two domains, the domains approach each other to close the cleft (Figure 4a). However, unlike *CesT*, in *RBP* each domain is discontinuous with respect to the sequence. The hinge that connects the two domains is made of three separate stretches of sequence (Figure 4b and Table 2). Consequently, the hinge torsion angles must change in a coordinated way, to prevent the two domains to disintegrate. Although violating the integrity of domains is energetically unfavorable, a lot of running time may be consumed on sampling non-favored conformations that disintegrate the domains. Indeed, when we simulated the motion of *RBP* without any external constraints, the domains wobbled and partially disintegrated during the motion, with high energy fluctuations (results not shown). Although this type of motion cannot be negated completely, domain disintegration during relatively fast substrate binding motion contrasts basic biological intuition.

In order to enforce loop closure, we have simply added a partial information predicate that penalizes disintegration of the domains (in terms of RMSD to the native domain). In the resulting pathway (Video S3), the two domains are kept in one piece throughout the motion, and only a small β-strand at the C-terminus of the protein (residues 266–269) deforms during the motion. The simulation of the motion in centroid mode is performed within a few minutes time. This demonstrates the flexibility of the partial information framework to efficiently address diverse settings, without the need for explicit *ad-hoc* calculations.

### The Degrees of Freedom that are Involved in the Motion of Cyanovirin-N

We now examine in detail the importance of different degrees of freedom for another model system of domain swapping motion: *Cyanovirin-N* is an anti-viral fusion inhibitor protein that binds to viral sugars, and is trialed for preventing sexual transmission of HIV. It comprises two repeat domains of 30% sequence identity. The domain swapped dimer has higher anti-viral affinity than the monomer [72], and it was shown that the two forms can exist in solution, with a high energy transition barrier between them. In addition, it has been reported that certain mutations can affect the energy barrier and stabilize alternative conformations [73]. We examined here how two repeat domains of a single chain can unpack from the tightly-intertwined monomeric conformation to an extended domain-swapped conformation. The conformational transition during swapping is substantial, as the swapped conformations deviate by 14 Å RMSD.

In all simulations, we started from the monomer conformation (pdb-id *2ezm* [74]), and for biasing the motion towards the swapped conformation (pdb-id *1l5e* [73]), we minimized the RMSD distance towards it. The difference between the following simulations is the sets of degrees of freedom that are allowed to rotate during the motion (Table 2).

#### The central hinge: Allowing rotation in residues 48–55

It has been suggested that residues 48–55 between the two repeat domains of *Cyanovirin-N* form a hinge region for domain swapping [72],[73]. Additional support comes from structural conservation patterns, difference in torsion angle values between alternative structures and Gaussian Network Models for detecting hinge regions (Table 2). We refer to this region as the *central hinge* of *Cyanovirin-N*.

Using the *central hinge* set of *dof*s (Table 2), we first used the simplified centroid mode representation to generate a low-energy motion pathway within minutes. Considering the experimentally determined high energy barrier for this motion, it is rather surprising that such a pathway could indeed be easily created. We thought this might be an artifact of the simplified representation of the structure in centroid mode: the barrier might be apparent only at a higher resolution level. We therefore proceeded to a full-atom representation:, When all side-chains atoms and hydrogen atoms were modeled explicitly, it was impossible to unlock the intertwined monomer, unless the energy threshold was substantially raised to 10^5^ Rosetta Score-12 units (which allows for extreme steric clashes). The domains did not unpack even in a long run of RRT, consisting of 300,000 conformations and taking a few days to run. The protein moved by no more than 1.5 Å from the initial conformation, over 13 Å away from the swapped target conformation. Video S4 demonstrates how the side-chains of one domain are tightly locked within the other domain, and the motion is confined within a steric “cage”.

#### The effect of local energy minimization

Could very slight “breathing” motions of other degrees of freedom allow the domain-swapping of the protein? Local energy minimization involves slight changes (∼0.1–0.2) in *all* backbone and side-chain degrees of freedom and as such might suffice to alleviate local steric clashes of the sensitive full atom energy function (see Methods section for details). Indeed, in a new simulation with freedom of motion in the central hinge together with local energy minimization (*“Central-Hinge (M)”* in Table 2) we were able to generate a clash-free motion pathway in full-atom. Local minimization slowed down the rate of generating new nodes (3–8 fold), but allowed the initial unpacking of the domains after less than an hour run. It is striking to observe how minute structural breathing in all degrees of freedom can alleviate steric clashes and allow motion that was not possible when only the central hinge is mobile.

#### The effect of adding secondary hinges

We now examine if the introduction of several additional *dofs* may provide the simulation with sufficient “breathing” flexibility to allow the large scale motion of the hinge, even without energy minimization. We inspected the structure and located two symmetry-related loops at residues 36–40 and at residues 87–91 that connect the two β-sheets in each domain. These loops appear as “weak links” in the protein chain between the two sheets (see Table 2 for more reasoning behind this choice). The addition of flexibility in the two loop regions (*“Secondary-Hinges”* in Table 2) allowed our simulations to find low-energy clash-free motions in full-atom mode (Video S5), without energy minimization in all *dof*s.

#### Analyzing hinge residues by restricted sampling of all DOFs

We saw that restricted local energy minimization of all *dof*s, as well as the introduction of secondary hinges both enabled the domain swapping motion. In order to analyze the motion of all residues, we conducted another simulation where the central hinge (residues 48–55) is free to move, and all other *dof*s can also rotate by up to 30° from their initial value, allowing for more extensive “breathing” motion in all *dof*s *(“Partially-Restricted (M)”* in Table 2 *)*. The total number of *dof*s in this simulation is 198, with 16 *dof*s that are completely free to move. Using this large number of *dof*s (and including local energy minimization), the simulations took 3–4 days. A movie of such a simulation (see Video S6) shows the breathing motion of all *dofs*, and in addition suggests that side-chains L1 and W49 (marked in red) act as “gate-keepers” that interfere in the unpacking of the two domains. It would be interesting to examine the role of these residues experimentally and *in-silico*, although this is out of the scope of this work.

We should note that the S–S bonds between two adjacent β-strands, from C8 to C22, and from C58 to C73, were not modeled in the simulation due to technical limitations. However, we note that both of these bonds connect adjacent β-strands, and atomic distances between these pairs of residues are close to constant during all the simulation, suggesting that S–S bonds will not play a critical role.

#### Consistency of the hinge regions between runs

**Figure 5:**
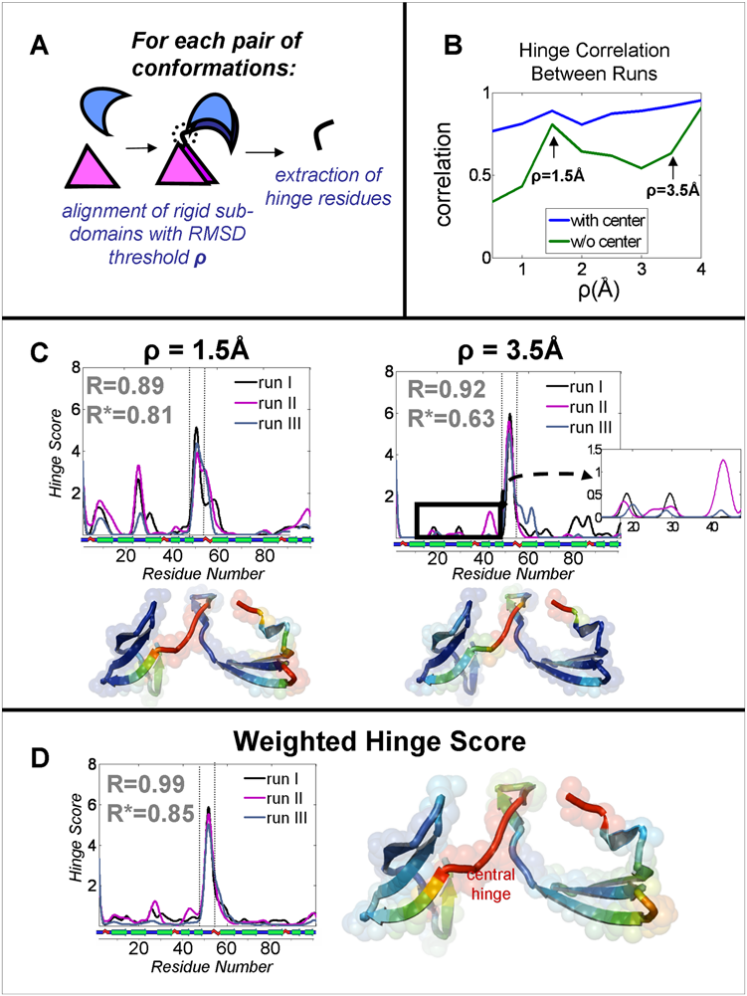
Hinge regions in independent simulations of *Cyanovirin-N* domain swapping. All runs use the *Partially-Restricted (M)* set of *dof*s (Table 2), where the central hinge is allowed free motion, and all other residues can rotate by ±30°. (A) In each run, each residue is scored by how often it tends to be in hinge regions. Hinges are extracted by structural comparison between conformations along the motion sequence. They connect sub-domains that remain rigid during the simulation. Resolution parameter ρ states the RMSD threshold used for rigid alignments. Mild hinges appear only at higher resolutions (low value of ρ), and salient hinges appear at low resolutions (see Figure S2 for a detailed protocol). (B) Pearson's Correlation between the hinge scores of three independent simulations, for different values of ρ. In blue, the correlation over all residues, including the central hinge (residues 48–55). In green, the correlation when excluding the central hinge. (C) Hinge scores for each residue in three independent simulations, for ρ = 1.5 Å and ρ = 3.5 Å. The y-axis denotes how often each residue appears in hinge regions (see Figure S2 for more details). Secondary structures (according to DSSP [86]) are plotted along the x-axis. Observe that milder hinges disappear at lower resolution (3.5 Å). R and R* are the average Pearson's correlations between runs with and without the central hinge region, respectively. For each plot, the crystal structure of *Cyanovirin-N* is colored according to the corresponding hinge score, with warm colors indicating higher scores. (D) The weighted average of the hinge scores for different values of ρ (see Results). Since higher resolutions contain milder hinges, they were assigned a lower weight.

In order to examine the residues involved in the motion, we have scored each residue for how often it serves as a hinge during the motion, at different resolutions of motion (see Methods and Figure S2). Low resolution hinges are involved in strong hinge motions, and high-resolution hinges are involved in milder ones. We conducted three independent simulations, and compared the consistency of the detected hinges at different resolutions of hinge motion (parameter ρ). We used Pearson's linear correlation (with values ranging between 1 for full-linear correlation, and 0 for no correlation). The correlation over all residues is very high and rises with decreasing resolution, such that the most prominent hinge motions are consistent between runs (Figure 5b, blue line; correlations range from 0.77 at ρ = 0.5 Å, to 0.96 for ρ = 4 Å). Since the central hinge is inherently biased by the run parameters, we also analyzed the correlation when excluding the central hinge region (green line). In this case the correlation is lower but still significant. As expected, the correlation is low at the highest resolution (ρ = 0.5 Å), where small, flickering, movements are measured. For a detailed inspection, we plot in Figure 5c the hinge scores in the three simulations at different resolutions (1.5 Å and 3.5 Å), and also show a *Cyanovirin-N* structure in cartoon representation colored based on the residue hinge score. Finally, a plot of the weighted average of hinge scores at different resolutions is shown in Figure 5d. Low-resolution hinges are involved in larger hinge motions and are assigned a higher weight, so that pronounced hinge regions are inspected:
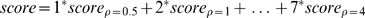



Not surprisingly, a large peak at the central hinge (marked in dashed lines in the plots) dominates in all figures. Also interesting are hinges in other regions, where restricted motion of ±30° was allowed. Flexibility in the tail region is apparent, although quite trivial. Interestingly, the hinges do not appear in random location, but rather are consistent between independent runs. For instance, residues 27–30 form an apparent hinge at resolution ρ = 1.5 Å, for all independent runs. At resolution of ρ = 3.5 Å the consistency seems less remarkable at first, but a focus on residues 10–50 shows very similar hinge patterns between runs (Figure 5c, inset).

#### Energy analysis

**Figure 6:**
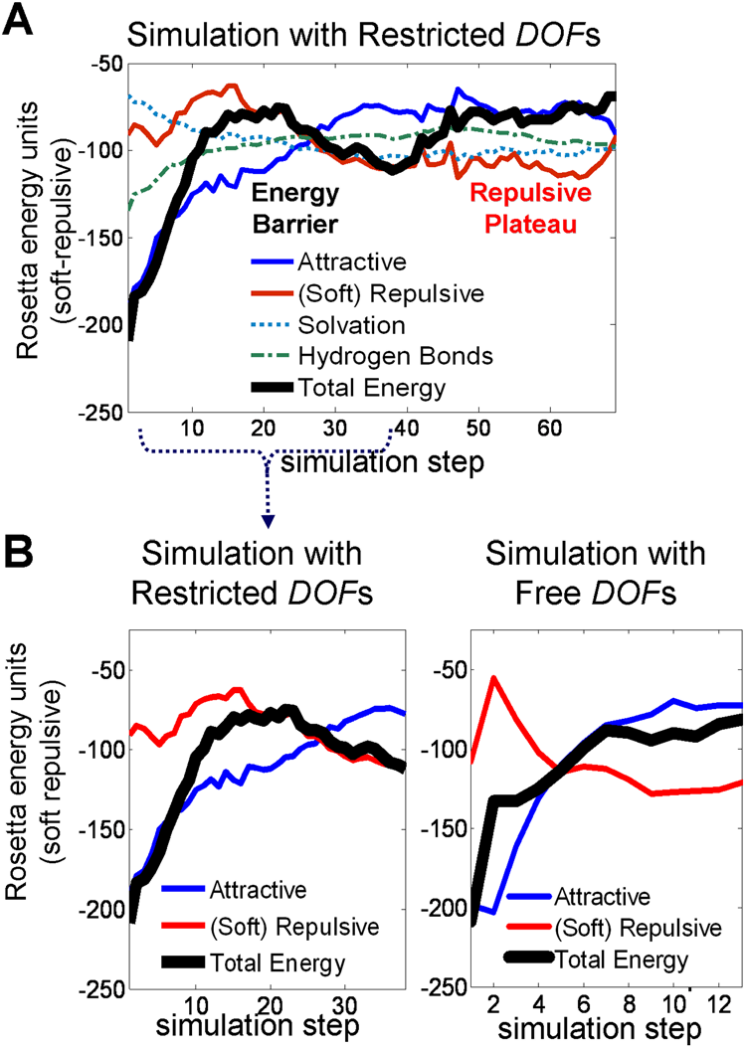
The contribution of different energy terms in the domain-swapping simulation of *Cyanovirin-N*. We used the Rosetta soft-repulsive energy score for this analysis, in order to dampen repulsive fluctuations that are due to mild sterical clashes (see text, note that the simulation itself was conducted with the Rosetta score-12 energy function, with a classical vdW potential). Note that the y-axis shows the total energy score, and the specific energy terms are shifted by a constant number of units, for convenient comparison with the other terms. (A) Energy plot for simulation with restricted *dof*s (central hinge is free to move, and all other *dof*s can fluctuate by ±30°). (B) Energy comparison for the aligned sections of the restricted (left) and free (right) simulations. In the free simulation, all *dof*s are free to move. The reaction coordinates of the two simulation were aligned by a string matching algorithm [39] based on structural similarity between conformations (see text, Figure S3 and Video S8).

What is the contribution of different energy terms to the motion? For analyzing the estimated energy landscape of the motion, we used the Rosetta energy score with dampened van der Waals potential [75], to reduce the dominant effect of fluctuative contributions of slight steric clashes (in the simulations themselves we use the classic van der Waals potential, so any steric clashes are heavily penalized). In Figure 6a we observe the energetic barrier that results from unpacking of the two domains, both disrupting favorable attractive forces (vdW–blue, hydrogen bonds - green) and causing increased repulsion due to the motion in a cluttered environment (repulsive force, red line). The solvation term decreases as polar residues are exposed (the hydrophobic effect is evident in the much higher increase in the attractive vdW force).

The repulsive forces subside after the domains have separated (steps 30–69 on the x-axis). Now that the two domains are unlocked, they may be free to sample many conformations without significant clashes. Indeed, there are several alternative conformations of domain swapped Cyanovirin-N [73].

#### Comparison of restricted and free simulations

Is the biasing for the central hinge indeed justified? In order to answer this question, we performed a simulation where all residues (except for the first and the last residue) were completely flexible and no bias was introduced (“*Free”* in Table 2). For 198 free *dof*s, the simulation took 5–6 days to generate a tree of 100,000 conformations. For the free simulation, the domains unpacked from each other substantially, but did not manage to reach the target conformation within the limitations of the running time (Video S7), probably due to the large number of non-restricted *dof*s.

**Figure 7:**
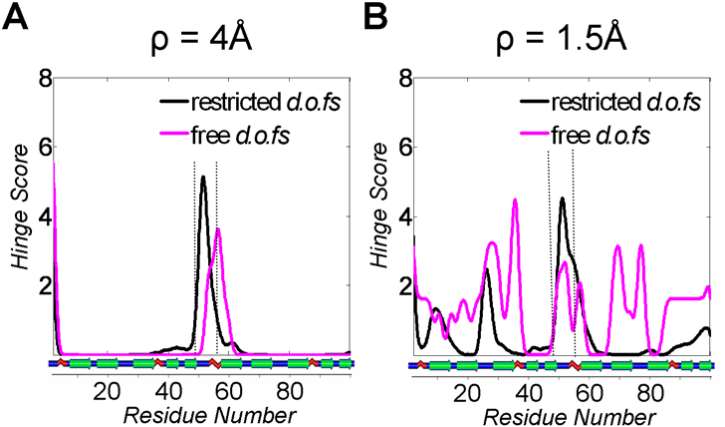
Robustness to restriction of *dof*s in *Cyanovirin-N* simulations. The hinge score for each residue is plotted for (A) restricted simulations, where only the central hinge (residues 48–55) is free to fully rotate, and the other residues are restricted to ±30° deviation from the initial conformation, and (B) a free simulation (magenta) where all backbone degrees of freedom are free to rotate (see Table 2). Hinge scores are plotted for resolution parameter values ρ = 4 Å and ρ = 1.5 Å. The central hinge is the most salient feature in the free simulation, and therefore it appears even in low-resolution plots. Milder hinges are less robust to the restriction of *dof*s (see text for more details).

We compared the free simulations to the “*Partially-restricted”* simulations described above and in Table 2. Each simulation results in a motion pathway that comprises a sequence of conformations. We aligned the simulated motion pathways based on RMSD between corresponding conformations (using a path alignment scheme where corresponding frames in the two simulations are aligned by a string matching algorithm, similar to sequence alignment methods, see [39]). The movie of the aligned motion of the restricted and free simulations (Video S8) demonstrates that the two simulations are very similar, and Cα RMSD between aligned conformations stays within 2–3 Å throughout most of the motion, growing to 3.5–4 Å only towards the end (Figure S3). Remarkably, comparison of the hinge scores in the restricted and free simulation (Figure 7), shows that the central hinge is the most prominent hinge at low resolution (left panel, ρ = 4 Å), which means it is involved in the largest scale motion in the free simulation. Milder hinge motions at resolution ρ = 1.5 Å are less correlated, although the hinges at residues 27–30 are still markedly observed in both simulations. Note that since the free simulation spanned a part of the domain swapping motion, the alignment is partial, comprising half of the partially-restricted simulation, and the entire free simulation.

In Figure 6b we also compare the energy of the aligned simulations. In both simulations we observe the energetic barrier that results from unpacking of the two domains, both disrupting favorable attractive forces (blue) and causing increased repulsion due to the motion in a cluttered environment (repulsive force, red line). In both simulations the repulsive forces subside as the domains continue to separate and the attractive forces increase.

It is worth noting that motion-planning with such a large number of *dof*s (198) is not a trivial task, and that both simulations converged only when we used local energy minimization. Although energy minimization may often increase the running time, it may allow to deal with a larger number of flexible *dof*s, since unlikely random perturbations that lead to clashes can be countered by local minimization.

## Discussion

In this work we showed how different types of partial information can be incorporated into the Rapidly-exploring Random Tree (RRT) algorithm. We present PathRover as a comprehensive framework, implemented within the Rosetta modeling infrastructure. In structural biology, partial information constraints are widely used in predictions of static minimal-energy conformations [47],[48],[76] and in MD simulations. The novelty in this work is the systematic introduction and the integration of partial information to sampling-based motion planning of molecules. In this sense, sampling based methods like RRT pose a natural framework for integrating prior biological information. From the perspective of algorithmic robotics, partial information is employed through a branch-termination scheme which is somewhat different from explicitly biasing the sampling of new conformations, used in previous works [25],[42],[43],[46]. This allows for the use of very general features, whereas biased sampling may require *ad-hoc* computations of a biased distribution functions that differ between various types of information.

We incorporated partial information into simulations of three different systems: *CesT* type III secretion chaperone, *Ribose Binding Protein (RBP),* and *Cyanovirin-N* anti-viral protein. Our analysis demonstrates how partial information constraints limit the search in the vast space of possible motion pathways. These constraints are motivated by existing and novel experimental methods for measuring constraints over transient conformations, or by expert intuition. In turn, computational observations allow for further subsequent validation by introducing detailed predictions of the motion that can be validated by experimental methods. We showed that the energy function prevents an over-bias by the partial information constraints, in case our prior information is inexact. PathRover simulations allowed us to assess the contribution of different residues to motion. Apparently, modest motions in specific regions may facilitate large-scale motions. The results from different simulations produced consistent patterns, and may therefore justify partial restriction of motion to improve running times. In particular, restricted and free simulations resulted in similar patterns of motion.

An important aspect of PathRover is its full embedding into the Rosetta modeling framework. Rosetta has repeatedly demonstrated an exceptional ability to produce high-quality results for a variety of different modeling tasks in the field of protein modeling, docking, protein design and other modeling challenges at atomic-level detail (e.g., [50]–[53]). The incorporation into Rosetta provides well-calibrated energy functions (both for centroid and full-atom simulations), efficient energy calculations, and a battery of established conformational sampling protocols. It also allows extension to additional predicates of partial information that were previously implemented in Rosetta, such as NMR coupling measurements and docking interface constraints. These have been used to guide and filter Rosetta Monte-Carlo searches, and will here be incorporated into RRT-based motion prediction.

### A Knowledge-Based Energy Function

Previous applications of the RRT algorithm have mainly been based on geometric considerations of clash avoidance or Van der Waals terms of established force fields. In some cases, more sophisticated terms were employed [32],[34]. Here we introduce the established Rosetta full-atom energy function into sampling based methods. Hence, we are able to generate motion pathways for complex movements that are at the same time energetically favorable and that abide by possibly known constraints about the motion. The full-atom energy function of Rosetta (we used here score12 [50]) includes physical terms such as van der Waals potential and solvation terms, as well as statistical knowledge based terms like the Ramachandran score, rotamer likelihood, statistical hydrogen bonding term and a simplified electrostatic score [49]. In some cases we observed that the repulsive energy term dominates the motion pathway: in a cluttered environment, clash avoidance is indeed probably the main contribution. Naturally, however, additional energy terms will affect the details of the motion pathways, such as solvation effects and electrostatic interactions [77]. We note that the statistical terms in Rosetta have straightforward interpretation in terms of physical properties. For instance, the Ramachandran score and rotamer likelihoods reflect steric hindrance in disallowed regions. The hydrogen bonding term was also shown to correlate remarkably with quantum-mechanical calculation [64]. While the original Rosetta energy function was optimized for native conformations, we postulate that it can also be used for the generation of clash-free, reasonable motion paths, which also account for other physical principles. Comparison to other common force-fields like CHARMM [78] and Amber [79] will provide additional credibility to PathRover simulations. In principle, PathRover is not restricted to any energy scoring function, as the energy scoring is a “black-box” in the implementation of the algorithm. As molecular mechanics energy functions are currently being added to the Rosetta modeling framework, we intend to compare different energy scoring functions in future work.

### Future Applications

#### Experimental validation and analysis of simulations

One of the big challenges of computational biology is the interface between computational and experimental observations. While full-atom experimental motion pathways of high resolution are still not in sight, significant progress has been recently made in experimental research of transient conformations. Distance constraints from FRET experiments, Paramagnetic-Resonance Enhancement, Residual Dipolar Coupling and other spectroscopic methods for assessing molecular dynamics can be used for (1) constraining simulations of molecular motion using measurable constraints, and for (2) validating motion pathways of suggested simulations, by comparing the measured distance constraints to simulated predictions. Our vision is that innovative experimental measurements of limited scope can focus and enhance computational techniques, effectively allowing researchers to generate realistic motion pathways that incorporate as much external information as possible within the currently suggested framework of PathRover. Particularly, this can allow for the design of experiments that target specific states within a motion pathway based on in silico predictions of large-scale motions. The predicted motions can be also used to suggest mutations, such as our suggested mutations in L1 and W49 for Cyanovirin-N (see residues marked in red in Videos S6 and S7).

Computational observations are most meaningful when they are well-defined in a way that poses them as clear, lab-testable hypotheses. To that end, it is not sufficient to rely on raw simulations results. In our work, we have therefore devoted significant effort for developing analysis and visualization tools for extracting physical features from simulated motion, including the protocol for analyzing hinge regions in simulated motion, as well as the visualization and space-time alignment of multiple motion paths. We believe that developing novel analysis and visualization tools is an important direction of future research, which is just as important as the simulations themselves, as it can provide the missing link between experimental and computational observations.

#### Applications to other types of molecular motion

In this work PathRover was applied to motions of domain swapping and substrate binding. However, different types of molecular motions might have different characteristics with respect to the number of torsion angles that are involved in the motion, the scale of the motion, the role of side-chains, etc. One challenging class of molecular motions involves allosteric protein motions [80]. In this case, a large number of torsion angles are often involved in the motion, but each of them changes by rather small increments, and partial information might constrain the overall nature of the motion. Another interesting type of motion involves more than one molecule, such as docking of a protein or a flexible peptide onto another protein. Motion-planning techniques have been used for small-molecule docking [40], but to the best of our knowledge not for docking of two globular proteins or for protein-peptide docking. Of particular interest within this framework are cases where partial information can provide details about the approximate location of the interface, and conformational backbone flexibility of the monomer needs to be modeled efficiently [71],[81].

#### Analyzing multiple motion pathways

One of the advantages of RRT-based techniques is their relative speed. A large body of motion pathways can be created at atomic level that includes side-chain atom positions. A large number of pathways provide further insights about the connectivity of the conformational space under a wide range of settings. In contrast, it is difficult to generate a large number of pathways using, e.g., MD simulations, due to slower running times. In Video S8 we showed an alignment between two motion pathways. We recently proposed a method to compare, cluster and merge multiple motion pathways from independent runs of the RRT algorithm. The merged pathways have lower energy or shorter length than all input pathways [39]. It would be interesting to examine the clusters of pathways that are generated with different types of partial information.

### Conclusions

This study proposes PathRover as a general and flexible setup where molecular systems can be explored, and constraints can be incorporated in a general and straightforward manner. Partial information can improve the performance of sampling based algorithms, by narrowing down the search in the vast conformational space of proteins. This is demonstrated in the present study on a number of molecular motions of specific interest. Future work will concentrate on refining protocols for additional systems and types of motions.

Beneficial crosstalk between experimental procedures and *in silico* simulations will ultimately optimize the wide integration of partial information into fast sampling-based algorithms–and forward our general understanding of protein motion and function.

## Supporting Information

Figure S1 Structural alignment between the pseudo-monomer of CesT (cyan) and its distant homologue SigE (red).
(0.93 MB TIF)

Figure S2 Protocol for hinge analysis of a motion path by structural comparison between conformations.
(0.75 MB TIF)

Figure S3 RMSD for alignment between restricted and free simulations throughout the simulation. The first half of the restricted simulation is aligned against the entire free simulation.
(0.14 MB TIF)

Table S1 Results of biasing the motion of *CesT* towards its distant homologue SigE with five different types of partial information
(0.05 MB DOC)

Text S1 PathRover Parameters List and Pseudo-Code for the *RRT_PARTIAL_INFO* Algorithm with Branch Termination
(0.50 MB DOC)

Video S1 Full-atom simulation of CesT starting from a domain swapped conformation. The pseudo-monomer is used as the partial information predicate to guide the motion.
(1.74 MB MPG)

Video S2 Simulation of *CesT* starting from a domain swapped conformation in centroid mode. The pseudo-monomer is used as the partial information predicate to guide the motion.
(1.21 MB MPG)

Video S3 Simulation of Ribose-Binding Protein motion.
(0.35 MB MPG)

Video S4 Simulation of *Cyanovirin-N* motion where only the central hinge is allowed to rotate and local minimization is not used.
(0.40 MB MPG)

Video S5 Simulation of Cyanovirin-N motion where both the central hinge and secondary hinges are allowed to rotate
(0.87 MB MPG)

Video S6 Partially restricted simulation of *Cyanovirin-N* motion where the central hinge is free to move, and all other residues can fluctuate by ±30°
(2.58 MB MPG)

Video S7 Free simulation of *Cyanovirin-N* motion where all residues are free to move.
(1.87 MB MPG)

Video S8 An alignment of the free and the partially-restricted simulations of *Cyanovirin-N*
(1.48 MB MPG)

## References

[1] Perutz MF, Rossman MG, Cullis AF, Muirhead H, Will G (1960). Structure of haemoglobin: a three-dimensional Fourier synthesis
at 5.5 Å resolution, obtained by X-ray analysis.. Nature.

[2] Kendrew JC, Dickerson RE, Strandberg BE, Hart RG, Davies DR (1960). Structure of myoglobin: a three-dimensional Fourier synthesis at
2 Å resolution.. Nature.

[3] Monod J, Wyman J, Changeux JP (1965). On the nature of allosteric transitions: a plausible model.. J Mol Biol.

[4] Gerstein M, Krebs W (1998). A Database of macromolecular motions.. Nucleic Acids Res.

[5] Berman HM, Westbrook J, Feng Z, Gilliland G, Bhat TN (2000). The Protein Data Bank.. Nucleic Acids Res.

[6] Cui Q, Bahar I (2006). Normal Mode Analysis: Theory and Applications to Biological and Chemical
Systems.

[7] Tobi D, Bahar I (2005). Structural changes involved in protein binding correlate with
intrinsic motions of proteins in the unbound state.. Proc Natl Acad Sci U S A.

[8] May A, Zacharias M (2008). Energy minimization in low-frequency normal modes to efficiently
allow for global flexibility during systematic protein-protein docking.. Proteins.

[9] Cammarata M, Levantino M, Schotte F, Anfinrud PA, Ewald F (2008). Tracking the structural dynamics of proteins in solution using
time-resolved wide-angle X-ray scattering.. Nat Methods.

[10] Henzler-Wildman K, Kern D (2007). Dynamic personalities of proteins.. Nature.

[11] Getz M, Sun X, Casiano-Negroni A, Zhang Q, Al-Hashimi HM (2007). NMR studies of RNA dynamics and structural plasticity using NMR
residual dipolar couplings.. Biopolymers.

[12] Lange OF, Lakomek NA, Fares C, Schroder GF, Walter KF (2008). Recognition dynamics up to microseconds revealed from an
RDC-derived ubiquitin ensemble in solution.. Science.

[13] Thirumalai D, Klimov DK (2007). Intermediates and transition states in protein folding.. Methods Mol Biol.

[14] Zhang Q, Stelzer AC, Fisher CK, Al-Hashimi HM (2007). Visualizing spatially correlated dynamics that directs RNA
conformational transitions.. Nature.

[15] Alder BJ, Wainwright TE (1959). Studies in molecular dynamics. I. general method.. J Chem Phys.

[16] McCammon JA, Gelin BR, Karplus M (1977). Dynamics of folded proteins.. Nature.

[17] Karplus M, Kuriyan J (2005). Molecular dynamics and protein function.. Proc Natl Acad Sci U S A.

[18] Henzler-Wildman KA, Lei M, Thai V, Kerns SJ, Karplus M (2007). A hierarchy of timescales in protein dynamics is linked to enzyme
catalysis.. Nature.

[19] Daggett V, Fersht A (2003). The present view of the mechanism of protein folding.. Nat Rev Mol Cell Biol.

[20] Feher VA, Cavanagh J (1999). Millisecond-timescale motions contribute to the function of the
bacterial response regulator protein Spo0F.. Nature.

[21] Isralewitz B, Gao M, Schulten K (2001). Steered molecular dynamics and mechanical functions of proteins.. Curr Opin Struct Biol.

[22] Yang LW, Rader AJ, Liu X, Jursa CJ, Chen SC (2006). oGNM: online computation of structural dynamics using the
Gaussian Network Model.. Nucleic Acids Res.

[23] Ueda Y, Taketomi H, Gö N (1978). Studies on protein folding, unfolding, and fluctuations by
computer simulation. II. A. Three-dimensional lattice model of lysozyme.. Biopolymers.

[24] Kavraki LE, Svestka P, Latombe J-C, Overmars MH (1996). Probabilistic roadmaps for path planning in high-dimensional
configuration spaces.. IEEE Trans Rob Autom.

[25] LaValle SM, Kuffner JJ, Donald BR, Lynch KM, Rus D (2001). Rapidly-exploring random trees: progress and prospects.. Algorithmic and Computational Robotics: New Directions.

[26] LaValle SM (2006). Sampling-based motion planning. Planning Algorithms.

[27] Hsu D, Latombe J-C, Motwani R, Latombe J-C (1997). Path planning in expansive configuration spaces.. IEEE International Conference on Robotics and Automation.

[28] Sánchez G, Latombe J-C (2003). A single-query bi-directional probabilistic roadmap planner with lazy
collision checking. Robotics Research.

[29] Hsu D, Kindel R, Latombe J-C, Rock S (2002). Randomized kinodynamic motion planning with moving obstacles.. Int J Robot Res.

[30] Choset H, Lynch KM, Hutchinson S, Kantor G, Burgard W (2005). Sampling-based algorithms. Principles of Robot Motion: Theory,
Algorithms, and Implementations.

[31] Latombe J-C (1999). Motion planning: a journey of robots, molecules, digital actors,
and other artifacts.. Int J Robot Res.

[32] Apaydin MS, Singh AP, Brutlag DL, Latombe J-C, Singh AP (2001). Capturing molecular energy landscapes with probabilistic
conformational roadmaps.. IEEE International Conference on Robotics and Automation.

[33] Amato NM, Song G (2002). Using motion planning to study protein folding pathways.. J Comput Biol.

[34] Amato NM, Dill KA, Song G (2003). Using motion planning to map protein folding landscapes and
analyze folding kinetics of known native structures.. J Comput Biol.

[35] Thomas S, Song G, Amato NM (2005). Protein folding by motion planning.. Phys Biol.

[36] Cortes J, Simeon T, Ruiz de Angulo V, Guieysse D, Remaud-Simeon M (2005). A path planning approach for computing large-amplitude motions of
flexible molecules.. Bioinformatics.

[37] Enosh A, Fleishman SJ, Ben-Tal N, Halperin D (2007). Prediction and simulation of motion in pairs of transmembrane
α-helices.. Bioinformatics.

[38] Kirillova S, Cortes J, Stefaniu A, Simeon T (2008). An NMA-guided path planning approach for computing
large-amplitude conformational changes in proteins.. Proteins.

[39] Enosh A, Raveh B, Furman-Schueler O, Halperin D, Ben-Tal N (2008). Generation, comparison and merging of pathways between protein
conformations: gating in K-channels.. Biophys J.

[40] Cortes J, Jaillet L, Simeon T (2007). Molecular disassembly with RRT-like algorithms. IEEE International
Conference on Robotics and Automation.

[41] Torrie GM, Valleau JP (1977). Nonphysical sampling distributions in Monte Carlo free-energy
estimation: umbrella sampling.. J Comput Phys.

[42] Zucker M, Kuffner JJ, Branicky M (2007). Multipartite RRTs for rapid replanning in dynamic environments. IEEE
International Conference on Robotics and Automation.

[43] Bekris KE, Kavraki LE (2007). Greedy but safe replanning under kinodynamic constraints. IEEE
International Conference on Robotics and Automation.

[44] Barraquand J, Latombe J-C, Latombe J-C (1990). A Monte-Carlo algorithm for path planning with many degrees of
freedom.. IEEE International Conference on Robotics and Automation.

[45] Kalisiak M, van de Panne M (2007). Faster motion planning using learned local viability models. IEEE
International Conference on Robotics and Automation.

[46] Zucker M, Kuffner JJ, Bagnell JA (2008). Adaptive workspace biasing for sampling-based planners. IEEE
International Conference on Robotics and Automation.

[47] Marti-Renom MA, Stuart AC, Fiser A, Sanchez R, Melo F (2000). Comparative protein structure modeling of genes and genomes.. Annu Rev Biophys Biomol Struct.

[48] de Vries SJ, van Dijk AD, Krzeminski M, van Dijk M, Thureau A (2007). HADDOCK versus HADDOCK: new features and performance of
HADDOCK2.0 on the CAPRI targets.. Proteins.

[49] Rohl CA, Strauss CE, Misura KM, Baker D (2004). Protein structure prediction using Rosetta.. Methods Enzymol.

[50] Qian B, Raman S, Das R, Bradley P, McCoy AJ (2007). High-resolution structure prediction and the crystallographic
phase problem.. Nature.

[51] Bradley P, Malmstrom L, Qian B, Schonbrun J, Chivian D (2005). Free modeling with Rosetta in CASP6.. Proteins.

[52] Kortemme T, Joachimiak LA, Bullock AN, Schuler AD, Stoddard BL (2004). Computational redesign of protein-protein interaction
specificity.. Nat Struct Mol Biol.

[53] Dantas G, Kuhlman B, Callender D, Wong M, Baker D (2003). A large scale test of computational protein design: folding and
stability of nine completely redesigned globular proteins.. J Mol Biol.

[54] Liu Y, Eisenberg D (2002). 3D domain swapping: as domains continue to swap.. Protein Sci.

[55] Janowski R, Kozak M, Jankowska E, Grzonka Z, Grubb A (2001). Human cystatin C, an amyloidogenic protein, dimerizes through
three-dimensional domain swapping.. Nat Struct Biol.

[56] Bennett MJ, Schlunegger MP, Eisenberg D (1995). 3D domain swapping: a mechanism for oligomer assembly.. Protein Sci.

[57] Yang S, Cho SS, Levy Y, Cheung MS, Levine H (2004). Domain swapping is a consequence of minimal frustration.. Proc Natl Acad Sci U S A.

[58] Esposito L, Daggett V (2005). Insight into ribonuclease A domain swapping by molecular dynamics
unfolding simulations.. Biochemistry.

[59] Yang S, Levine H, Onuchic JN (2005). Protein oligomerization through domain swapping: role of
inter-molecular interactions and protein concentration.. J Mol Biol.

[60] Malevanets A, Sirota FL, Wodak SJ (2008). Mechanism and energy landscape of domain swapping in the B1
domain of protein G.. J Mol Biol.

[61] Echols N, Milburn D, Gerstein M (2003). MolMovDB: analysis and visualization of conformational change and
structural flexibility.. Nucleic Acids Res.

[62] Shatsky M, Nussinov R, Wolfson HJ (2002). Flexible protein alignment and hinge detection.. Proteins.

[63] Kuhlman B, Baker D (2000). Native protein sequences are close to optimal for their
structures.. Proc Natl Acad Sci U S A.

[64] Morozov AV, Kortemme T, Tsemekhman K, Baker D (2004). Close agreement between the orientation dependence of hydrogen
bonds observed in protein structures and quantum mechanical calculations.. Proc Natl Acad Sci U S A.

[65] Davis IW, Arendall WB, Richardson DC, Richardson JS (2006). The backrub motion: how protein backbone shrugs when a sidechain
dances.. Structure.

[66] Luo Y, Bertero MG, Frey EA, Pfuetzner RA, Wenk MR (2001). Structural and biochemical characterization of the type III
secretion chaperones CesT and SigE.. Nat Struct Biol.

[67] Canutescu AA, Dunbrack RL (2003). Cyclic coordinate descent: a robotics algorithm for protein loop
closure.. Protein Sci.

[68] Cortes J, Simeon T (2004). Sampling-based motion planning under kinematic loop closure
constraints.. Algorithmic Foundations of Robotics VI.

[69] Xie D, Amato NM (2004). A kinematics-based probabilistic roadmap method for high DOF closed
chain systems. IEEE International Conference on Robotics and Automation.

[70] Kolodny R, Guibas L, Levitt M, Koehl P (2005). Inverse kinematics in biology: the protein loop closure problem.. Int J Robot Res.

[71] Wang C, Bradley P, Baker D (2007). Protein-protein docking with backbone flexibility.. J Mol Biol.

[72] Botos I, O'Keefe BR, Shenoy SR, Cartner LK, Ratner DM (2002). Structures of the complexes of a potent anti-HIV protein
cyanovirin-N and high mannose oligosaccharides.. J Biol Chem.

[73] Barrientos LG, Louis JM, Botos I, Mori T, Han Z (2002). The domain-swapped dimer of cyanovirin-N is in a metastable
folded state: reconciliation of X-ray and NMR structures.. Structure.

[74] Bewley CA, Gustafson KR, Boyd MR, Covell DG, Bax A (1998). Solution structure of cyanovirin-N, a potent HIV-inactivating
protein..

[75] Dantas G, Corrent C, Reichow SL, Havranek JJ, Eletr ZM (2007). High-resolution structural and thermodynamic analysis of extreme
stabilization of human procarboxypeptidase by computational protein design.. J Mol Biol.

[76] Schroder GF, Brunger AT, Levitt M (2007). Combining efficient conformational sampling with a deformable
elastic network model facilitates structure refinement at low resolution.. Structure.

[77] Boas FE, Harbury PB (2007). Potential energy functions for protein design.. Curr Opin Struct Biol.

[78] Brooks B, Bruccoleri R, Olafson B, States D, Swaminathan S (1983). CHARMM: a program for macromolecular energy, minimization, and
dynamics calculations.. J Comput Chem.

[79] Case DA, Cheatham TE, Darden T, Gohlke H, Luo R (2005). The Amber biomolecular simulation programs.. J Comput Chem.

[80] Daily MD, Gray JJ (2007). Local motions in a benchmark of allosteric proteins.. Proteins.

[81] Bonvin AM (2006). Flexible protein-protein docking.. Curr Opin Struct Biol.

[82] DeLano WL (2002). The PyMOL User's Manual.

[83] Björkman AJ, Mowbray SL (1998). Multiple open forms of ribose-binding protein trace the path of
its conformational change.. J Mol Biol.

[84] Björkman AJ, Binnie R, Zhang H, Cole L, Hermodson M (1994). Probing protein-protein interactions. The ribose-binding protein
in bacterial transport and chemotaxis.. J Biol Chem.

[85] Alexandrov N, Shindyalov I (2003). PDP: protein domain parser.. Bioinformatics.

[86] Kabsch W, Sander C (1983). Dictionary of protein secondary structure: pattern recognition of
hydrogen-bonded and geometrical features.. Biopolymers.

