# Supplementary figures and images for: Rapid Sampling of Molecular Motions with Prior Information Constraints
Source: PLoS Comput Biol. 2009 Feb 27;5(2):e1000295. doi: 10.1371/journal.pcbi.1000295 (PMC2637990; doi:10.1371/journal.pcbi.1000295)

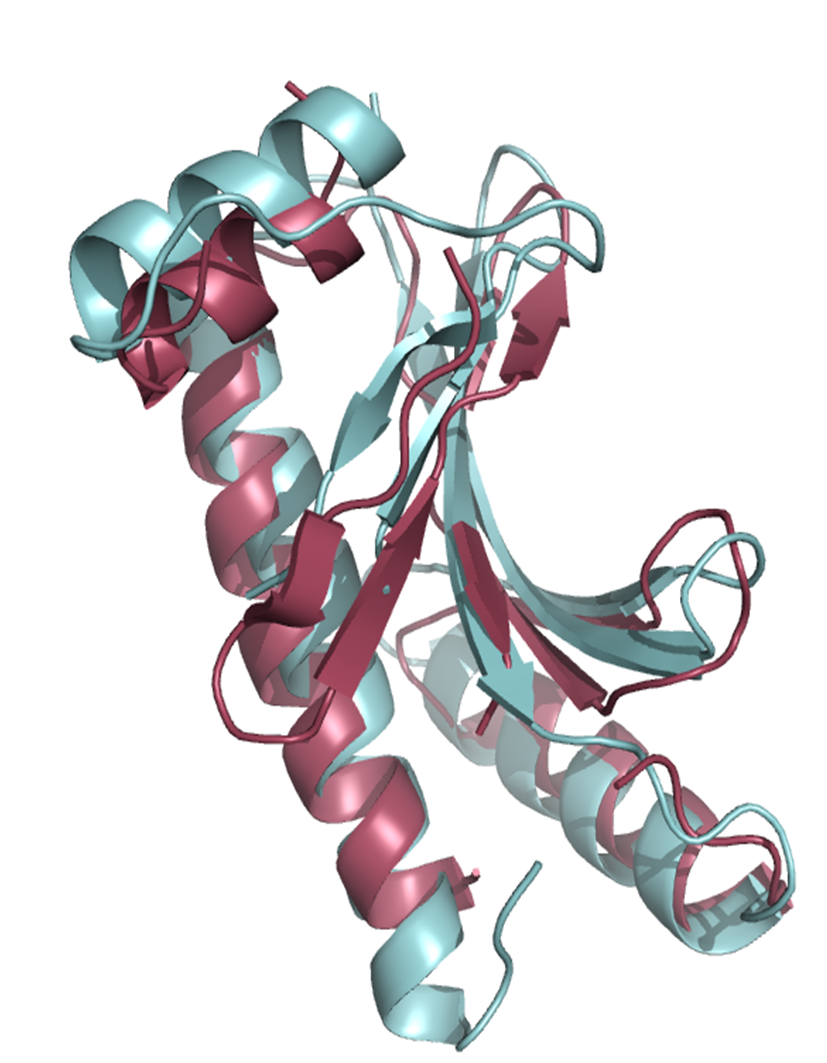

Supplement: Figure S1 — Structural alignment between the pseudo-monomer of CesT (cyan) and its distant homologue SigE (red). (0.93 MB TIF) [file pcbi.1000295.s001.tif]

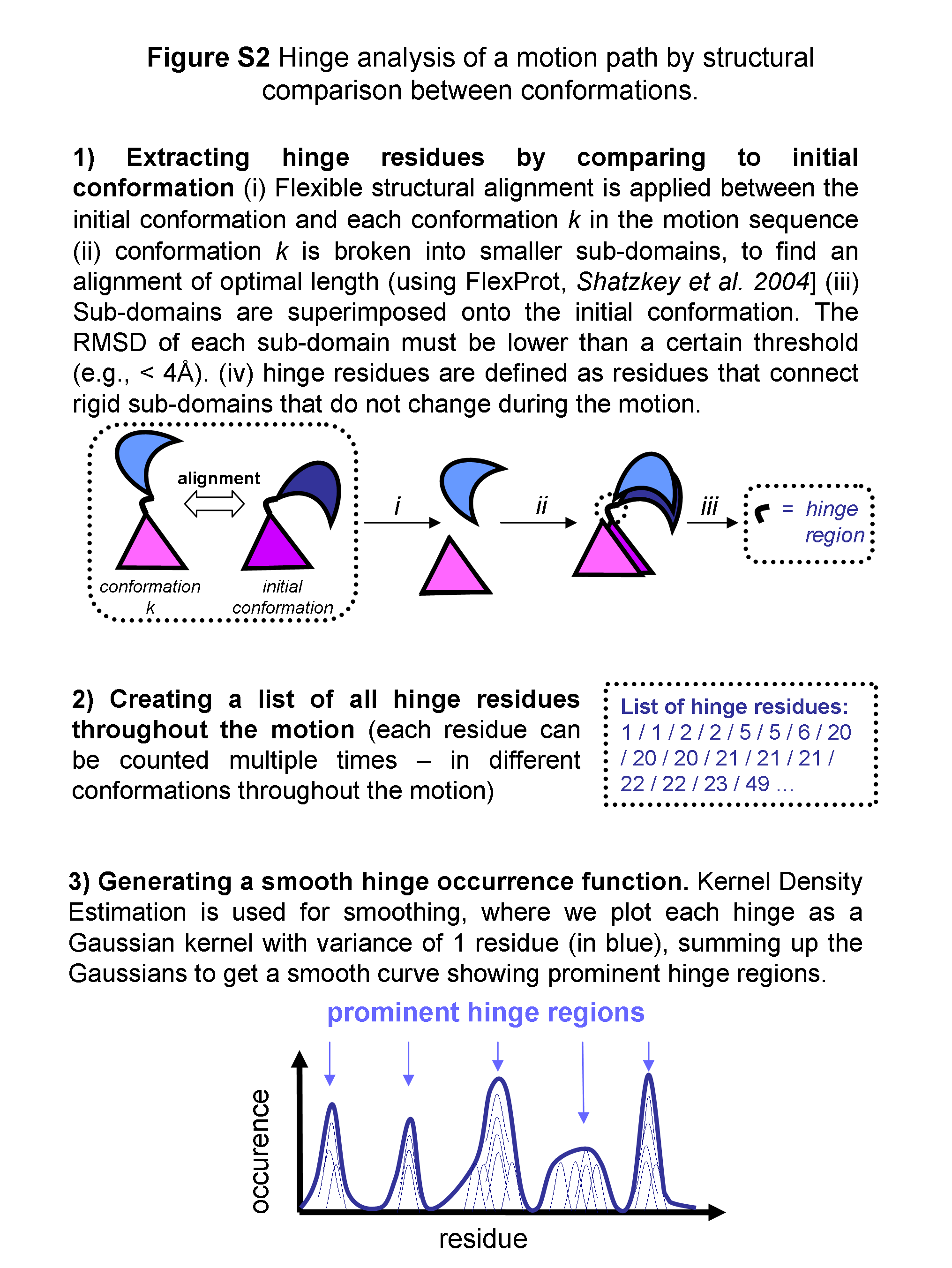

Supplement: Figure S2 — Protocol for hinge analysis of a motion path by structural comparison between conformations. (0.75 MB TIF) [file pcbi.1000295.s002.tif]

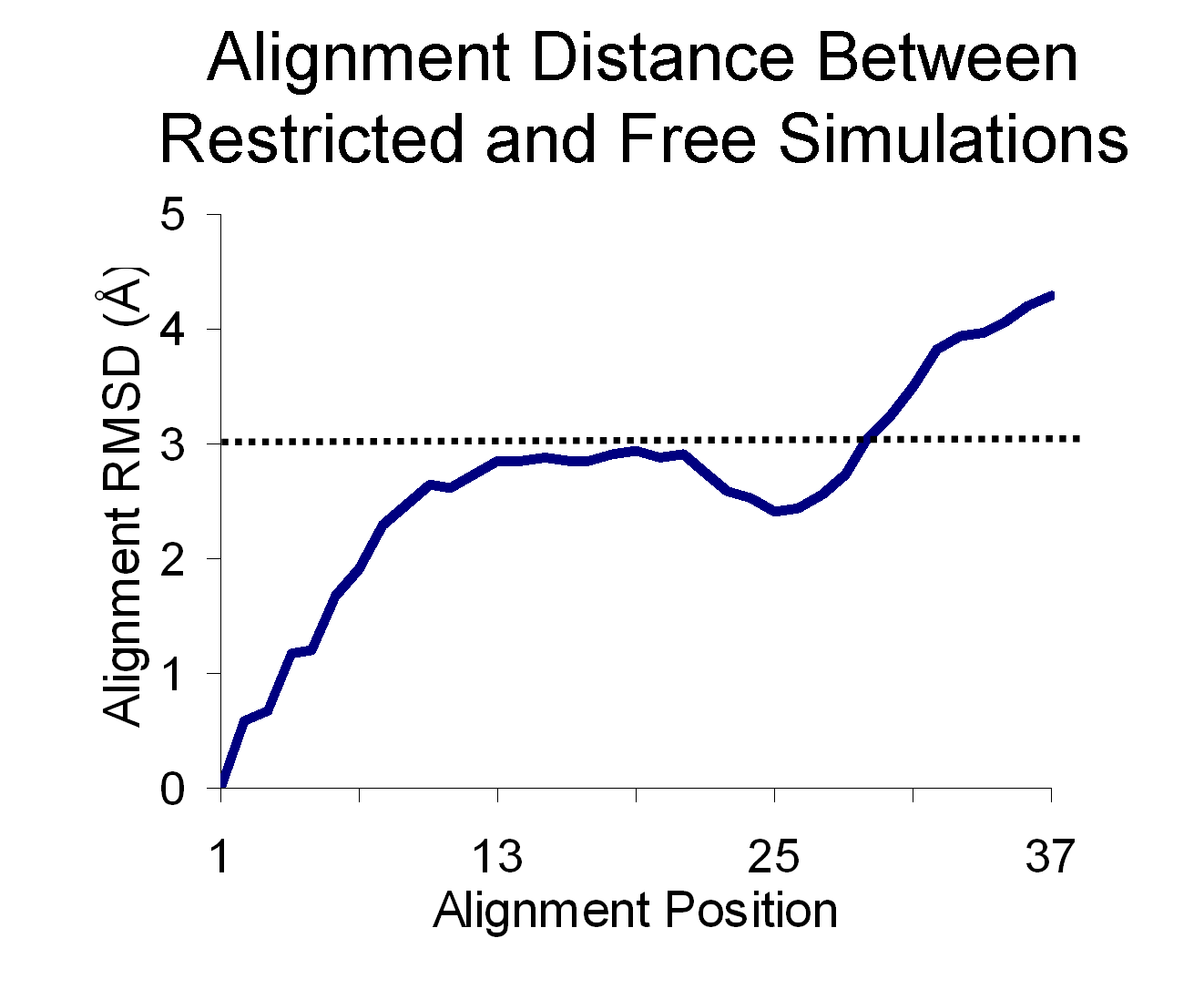

Supplement: Figure S3 — RMSD for alignment between restricted and free simulations throughout the simulation. The first half of the restricted simulation is aligned against the entire free simulation. (0.14 MB TIF) [file pcbi.1000295.s003.tif]
