# Supplementary material for: Rapid Sampling of Molecular Motions with Prior Information Constraints
Source: PLoS Comput Biol. 2009 Feb 27;5(2):e1000295. doi: 10.1371/journal.pcbi.1000295 (PMC2637990; doi:10.1371/journal.pcbi.1000295)
Supplement: Table S1 — Results of biasing the motion of CesT towards its distant homologue SigE with five different types of partial information (0.05 MB DOC) [file pcbi.1000295.s004.doc]

*† LLD / LLA = Least Mean Square Line Distance / Angle ; CMD = Center of Mass Distance*

*†† LLA measured in angles*

***pseudo-monomer***

***SigE***

**1.8**

**6.4**

**2.0**

**1.9**

**8.9**

***CMD***

***LLA^††^***

***LLD***

91°

279°

275°

11.1

15.0

47.2

13.8

7.8

13.8

25.1

39.1

**Start Conformation (*CesT*)**

***(v)***

***(iv)***

***(iii)***

***(ii)^†^***

***(i)***

***Partial Data***

***Evaluated on***

**3.2**

**8.2**

**3.6**

**5.3**

**6.7**

**RMSD to Final Conf.**

1.8

1.2

1.2

8.6

6.1

**Final Conformation**

0

0

0

8.8

6.1

**Target Conformation (*SigE*)**

**a**

**b**

**Legend for types of partial information:** The technical formulation of predicates is listed in Table 1, and illustrated in Figure 2b.

**(i)** *Pair-Distance* predicate between atoms (PHE12;LEU95) in *CesT* and (PHE12;GLU110) in *SigE*.

**(ii)** *Line-Distance*, *Line-Angle* and *Cent-Mass Distance* predicates between helices H1 and H3 in different domains of *CesT* and *SigE*.

**(iii) / (iv) / (v)** *Aligned RMSD-Match* predicate between pairs of secondary structures (α-helices: H1 and H3, β-strands:B0 and B1) in different domains of CesT and SigE: *(iii)* α-helices *(iv)* β-strands *(v)* α-helices + β-strands.

**Table S1** Results of biasing the motion of CesT towards its distant homologue *SigE* with five different types of partial information (see legend box, Table 3 and Figure 3 for more details). All values are given in Ångstroms, except where indicated. **(a)** Comparison of predicate evaluation on Start (*CesT*), Target (*SigE*) and Final conformation in the motion pathway. **(b)** RMSD between the final conformation and either *SigE*, or the *CesT* pseudo-monomer conformation (see Figure 3 for comparison). Although *SigE* was used to direct the motion, the final conformation is, in general, more similar to the *CesT* pseudo-monomer, except for one type of partial information (first column).
