# Supplementary material for: Rapid Sampling of Molecular Motions with Prior Information Constraints
Source: PLoS Comput Biol. 2009 Feb 27;5(2):e1000295. doi: 10.1371/journal.pcbi.1000295 (PMC2637990; doi:10.1371/journal.pcbi.1000295)
Supplement: Text S1 — PathRover Parameters List and Pseudo-Code for the RRT_PARTIAL_INFO Algorithm with Branch Termination (0.50 MB DOC) [file pcbi.1000295.s005.doc]

**Supplementary Text S1:** PathRover Parameters List and Pseudo-Code for
the RRT_PARTIAL_INFO Algorithm with Branch Termination

**I. PathRover - Parameters List:**

| **Parameter Name** | **Parameter Description** | **Default Value** |
| --- | --- | --- |
| **General Parameters:** | | |
| *MAX_TREE_SIZE* | Maximal number of nodes in the entire RRT tree | 30,000 |
| *MAX_CONSEC_FAILS* | Maximal number of consecutive failed iterations | 100 |
| **Energy Parameters:** | | |
| *ENERGY_FUNCTION* | The energy scoring function used for scoring the nodes (e.g., Rosetta full-atom or simplified centroid mode energy functions) | *The Rosetta default full-atom score (“score12”)* |
| *ENERGY_THRESHOLD* | The maximal energy score for valid conformations | 0.0 (centroid mode) / 450.0 (full-atom) |
| *USE_LOCAL_ENERGY _MINIMIZATION* | If true, a local energy minimization procedure is applied for each node before energy scoring, mostly in order to remove very slight steric clashes | TRUE |
| **Degrees of Freedom Parameters:** | | |
| *DOFS _LIST* | a list that specifies the backbone torsion angles (φ/ψ) that are free to change during the sampling of random nodes | *all backbone degrees of freedom* |
| *DOFS_ DEVIATION* | For each degree of freedom in *DOFS_LIST*, this parameter specifies the maximal deviation from the initial structure, during the sampling of random nodes | ±180° |
| **Extend-Tree Parameters (“local-planner”):** | | |
| *EXTEND_MAX _NUM_STEPS* | The maximal number of steps in a single invocation of the local planner | 15 |
| *EXTEND_MAX_STEP*  *_SIZE* | The maximal change of any torsion angle in a single step of the local planner, i.e., the largest value of any coordinate in the vector Δ in the local planner | ±1.5° |
| *MAX_STEPS_PARTIAL-*  *_INFO_NOT_IMPROVED* | the maximal number of consecutive nodes that the local-planner is allowed to add, without improving the partial info score | 2 |

**II. Pseudo-Code for the RRT_PARTIAL_INFO Algorithm with Branch Termination, as implemented in PathRover**

The input is an initial conformation q_init_. For incorporating partial information, we assume a continuous function over the conformation space, the “PARTIAL_INFO_SCORE”. This function serves as a general way for incorporating predicates of partial information. For each conformation *q*, this function evaluates how well *q* conforms to specified predicates of partial information constraints.


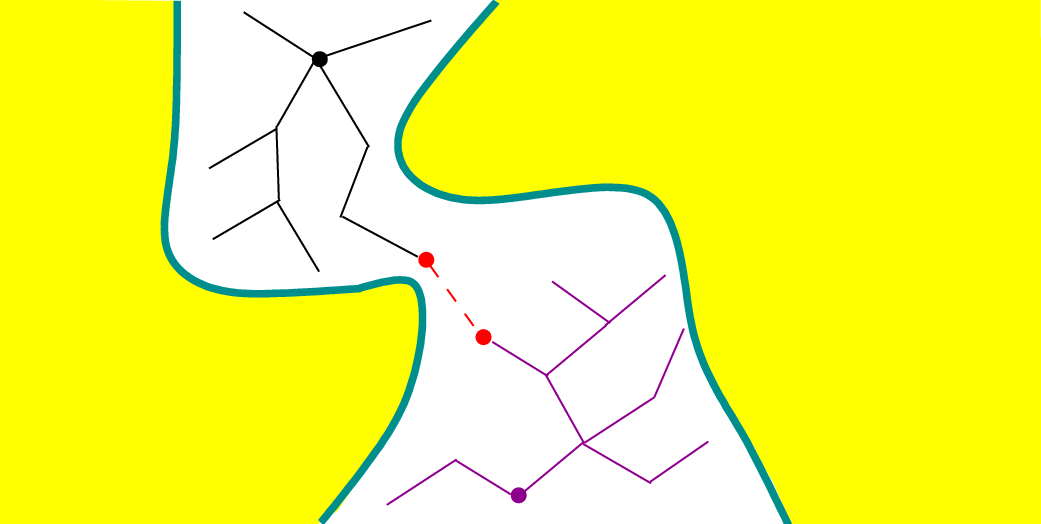


***Conformational space***

***q_init_***

***q_near_***

***q_goal_***

C_feasible_ – plausible

conformations

C_forbid_ – high energy conformations


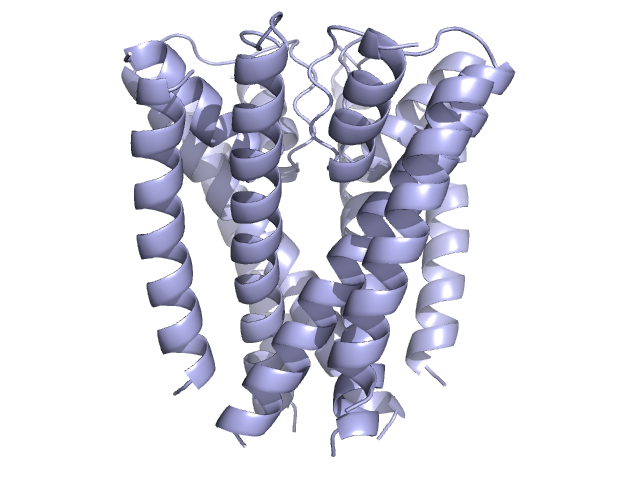

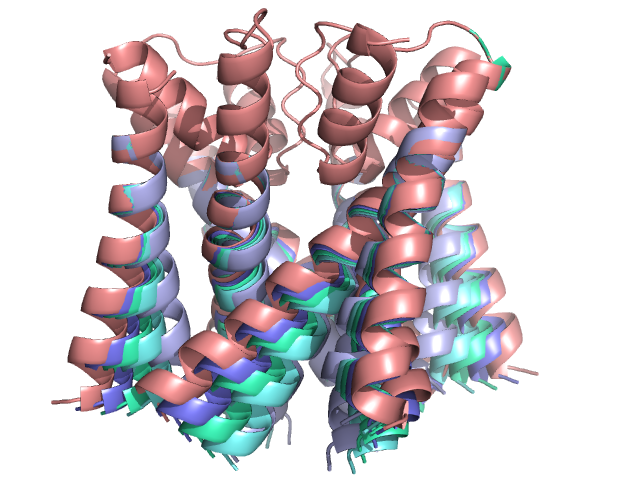


**Nodes** = conformations

**Edges** = motion in feasible space

**Illustration of the construction of an RRT tree.** The conformation space is divided into feasible and forbidden conformations. The tree is randomly expanded to cover the space of feasible conformations. The partial information predicate is used to terminate branches that do not adhere to or improve these predicates.

***q_rand_***

*Interpolating from* q_near_ to q_rand_

1. RRT_PARTIAL_INFO is the main procedure for building a tree of conformations: each node in the tree is a conformation, and each edge is some motion in the space of feasible (low-energy, clash-free) conformations (see figure above).

**RRT_PARTIAL_INFO ( q_init_ )**

*// to escape partial-info local-minima, partial info is used only every second iteration*

use_partial_info = TRUE

tree.ADD_NODE(q_init_)

While (tree.size < **[MAX_TREE_SIZE]**) and not STOP_CRITERIA() )

q_rand_ = SAMPLE_NODE( **[BACKBONE_DOF_LIST]** )

q_near_ = tree.NEAREST_NEIGHBOR( q_rand_ )

EXTEND_TREE_LOCALLY( q_near_ 🡪 q_rand_ , use_partial_info )

use_partial_info = not use_partial_info

End

1. EXTEND_TREE_LOCALLY is used to interpolate between nodes when extending the tree (referred to as the *local-planner* in algorithmic robotics). The energy score of each interpolated conformation is evaluated to restrict the tree to collision-free, low-energy motions. Deviating slightly from the typical description of RRT, we explicitly store interpolated samples in the tree, and not only the destination node, for simplicity of implementation. This does not affect the output of the algorithm, only memory requirements.

**EXTEND_TREE_LOCALLY ( q_from_ 🡪 q_to_ , use_partial_info)**

*// dofs_step* is a vector for changing the conformational degrees of freedom in each interpolation step

*// α* is a normalization factor that restricts the largest coordinate in *dofs_step* to parameter **[EXTEND_MAX_STEP_SIZE]**

dofs_step = α * [ dofs(q_to_) – dofs(q_from_) ]

q = q_from_

q­_next_ = q + dofs_step

For i = 1..[**EXTEND_MAX_NUM_STEPS]**

If ENERGY(q_next_) > [**ENERGY_THRESHOLD]** then

Break

If use_partial_info then

If not PARTIAL_INFO_IMPROVED( q ) then

Break

tree.ADD_EDGE( q 🡪 q + step_vector )

q = q + step_vector

End

1. PARTIAL_INFO_IMPROVED evaluates the partial information score, in order to terminate branches that do not improve the partial information predicates.

**PARTIAL_INFO_IMPROVED ( q ):**

If ( PARTIAL_INFO_SCORE( q ) did not improve for more than [**MAX_STEPS_ PARTIAL_INFO_NOT_IMPROVED]** consecutive steps )
 then Return FALSE

Return TRUE

1. STOP_CRITERIA is a global termination function.

**STOP_CRITERIA ( init_node )[[1]](#footnote-2)**

If tree.size > [**MAX_TREE_SIZE]** ) then

Return TRUE

If ( tree could not be expanded for more
than **[MAX_CONSEC_FAILS]** ) then

Return TRUE

1. In general implementations, the stop citeria can be used to save further running time by stopping when a certain conformation was reached (such as the target conformation), or if a desired partial information measure decreased below a threshold level. [↑](#footnote-ref-2)
